# Nandrolone decanoate administration does not attenuate muscle atrophy during a short period of disuse

**DOI:** 10.1371/journal.pone.0210823

**Published:** 2019-01-28

**Authors:** Astrid M. H. Horstman, Evelien M. P. Backx, Joey S. J. Smeets, Gabriel N. Marzuca-Nassr, Janneau van Kranenburg, Douwe de Boer, John Dolmans, Tim Snijders, Lex B. Verdijk, Lisette C. P. G. M. de Groot, Luc J. C. van Loon

**Affiliations:** 1 NUTRIM School of Nutrition and Translational Research in Metabolism, Maastricht University Medical Centre+, Maastricht, The Netherlands; 2 Department of Human Nutrition, Wageningen University, Wageningen, The Netherlands; 3 Department of Clinical Chemistry, Central Diagnostic Laboratory, Maastricht University Medical Centre+, Maastricht, The Netherlands; 4 Department of Surgery, Maastricht University Medical Centre+, Maastricht, The Netherlands; University of Sydney, AUSTRALIA

## Abstract

**Background:**

A few days of bed rest or immobilization following injury, disease, or surgery can lead to considerable loss of skeletal muscle mass and strength. It has been speculated that such short, successive periods of muscle disuse may be largely responsible for the age-related loss of muscle mass throughout the lifespan.

**Objective:**

To assess whether a single intramuscular injection of nandrolone decanoate prior to immobilization can attenuate the loss of muscle mass and strength *in vivo* in humans.

**Design, setting and participants:**

Thirty healthy (22 ± 1 years) men were subjected to 7 days of one-legged knee immobilization by means of a full leg cast with (NAD, *n* = 15) or without (CON, *n* = 15) prior intramuscular nandrolone decanoate injection (200 mg).

**Measures:**

Before and immediately after immobilization, *quadriceps* muscle cross-sectional area (CSA) (by means of single-slice computed tomography (CT) scans of the upper leg) and one-legged knee extension strength (one-repetition maximum [1-RM]) were assessed for both legs. Furthermore, muscle biopsies from the immobilized leg were taken before and after immobilization to assess type I and type II muscle fiber cross-sectional area.

**Results:**

*Quadriceps* muscle CSA decreased during immobilization in both CON and NAD (-6 ± 1% and -6 ± 1%, respectively; main effect of time *P*<0.01), with no differences between the groups (time × treatment interaction, *P* = 0.59). Leg muscle strength declined following immobilization (-6 ± 2% in CON and -7 ± 3% in NAD; main effect of time, *P*<0.05), with no differences between groups (time × treatment interaction, *P* = 0.55).

**Conclusions:**

This is the first study to report that nandrolone decanoate administration does not preserve skeletal muscle mass and strength during a short period of leg immobilization *in vivo* in humans.

## Introduction

Muscle disuse, such as forced upon following injury or during illness, can lead to substantial loss of skeletal muscle mass and strength and has numerous negative side effects. Most periods of muscle disuse tend to be of short duration, generally less than 7 days [1, 2]. Recently, it has been shown that even a few days of muscle disuse can lead to a substantial decline in both muscle mass as well as muscle strength [3]. It has been speculated that such short successive periods of bed rest or immobilization may be responsible for the greater part of muscle mass that is lost throughout the lifespan [1, 4]. Obviously, effective exercise, nutritional and/or pharmaceutical strategies are required to prevent or attenuate skeletal muscle loss during such short, successive periods of muscle disuse [5].

Skeletal muscle maintenance largely depends on the presence of two main anabolic stimuli, dietary protein intake and physical activity [6]. Maintenance of some level of physical activity is required to allow skeletal muscle mass preservation. However, this is not always possible in case of disease, injury, and/or acute hospitalization. Furthermore, during a short period of bed rest or immobilization, energy intake generally becomes compromised. As a consequence, dietary protein consumption is temporarily lowered thereby accelerating muscle loss [7]. Previous work has shown that maintaining habitual dietary protein intake will attenuate muscle loss [4, 8–11], but protein supplementation well above habitual protein intake levels does not preserve muscle mass during limb immobilization [12]. Therefore, adjuvant pharmaceutical interventions may be useful in preserving muscle mass during short periods of bed rest or limb immobilization.

Skeletal muscle disuse is accompanied by a decline in basal, post-absorptive muscle protein synthesis rates, an increase in protein breakdown, and the development of anabolic resistance to feeding [1, 13]. The anabolic androgenic steroid nandrolone, with nandrolone decanoate as one of its esters [14–17], has been shown to increase protein synthesis and decrease proteolysis [18, 19]. Prolonged nandrolone decanoate administration (ranging from 200–2400 mg for a period between 1–24 months) has been shown to increase fat free mass, muscle cross-sectional area (CSA) and/or strength in several pathological conditions *in vivo* in humans [20–32]. Based on these findings it could be speculated that nandrolone decanoate administration may represent an effective adjuvant pharmaceutical strategy to prevent or attenuate disuse atrophy.

Therefore, the objective was to assess whether a single intramuscular injection of nandrolone decanoate prior to immobilization can attenuate the loss of muscle mass and strength *in vivo* in humans. We hypothesize that intramuscular administration of a single dose of nandrolone decanoate attenuates muscle mass and strength loss during subsequent leg immobilization. To test this hypothesis we selected 30 healthy adults who were subjected to 7 days of one-legged knee immobilization. One group (*n* = 15) received a single intramuscular nandrolone decanoate injection (200 mg) immediately prior to immobilization, whereas the other group (*n* = 15) acted as controls. Skeletal muscle mass and function were assessed before and immediately after immobilization.

## Material and methods

### Subjects

**Fig 1:**
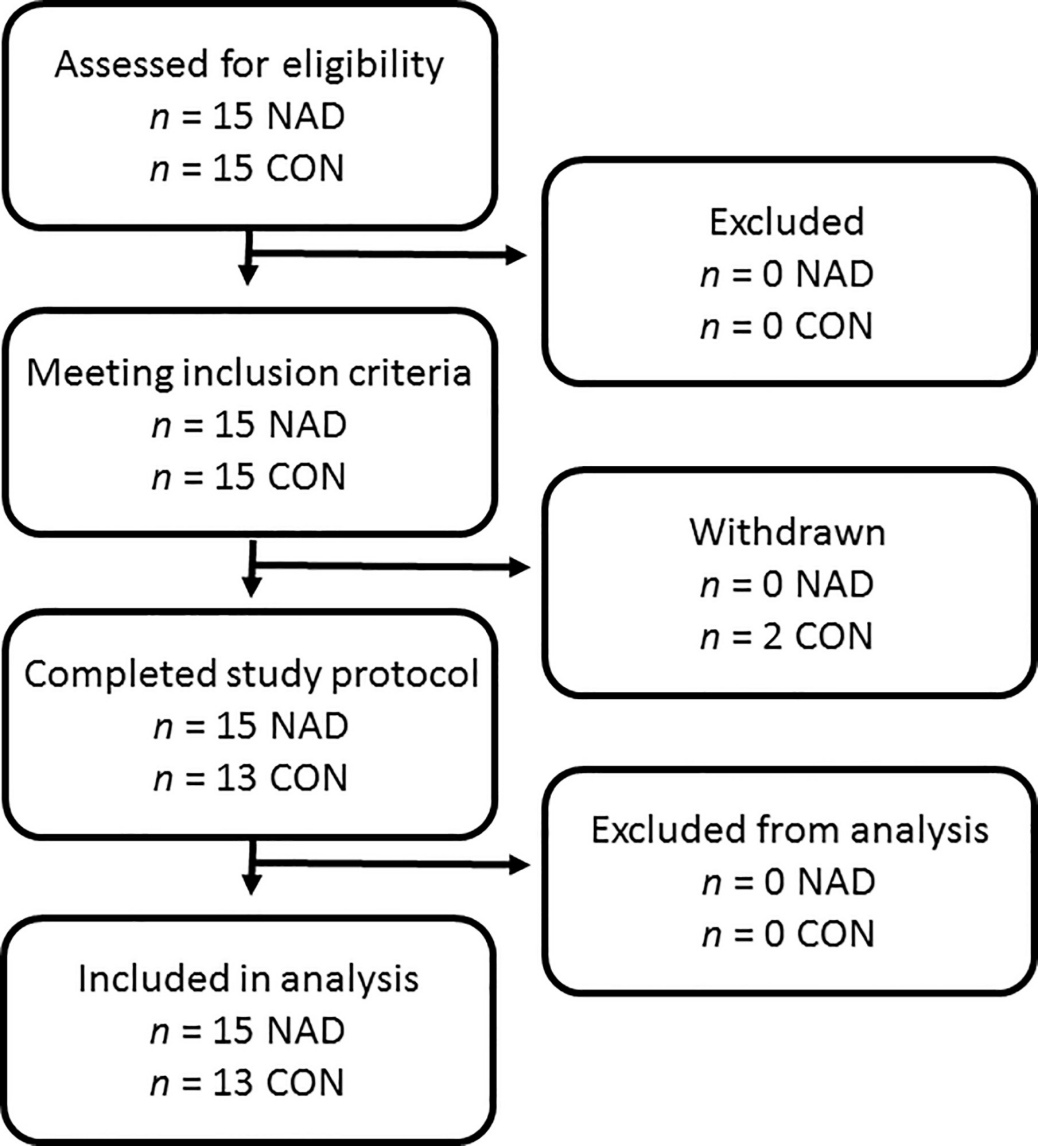
Flowchart of participants.

Thirty young (18–35 years) healthy males (18.5 < body mass index (BMI) < 30 kg/m^2^) participated in this experiment between October 2014 and April 2016 (Fig 1). Participants’ characteristics are presented in Table 1. All subjects were screened and excluded in case one or more of the following criteria were met: a history of thromboembolic events, smoker, history of participating in regular resistance type exercise training, back/leg/knee/shoulder complaints that could interfere with the use of crutches, systematic use of corticosteroids, anabolic androgenic steroids, growth hormone, immunosuppressants, insulin, or blood glucose lowering medication, pre-existing renal disease or potential risk for renal dysfunction (diabetes, hypertension, reduced glomerular filtration rate), liver disease, heart failure or migraines. The study was performed as part of a greater project in which we also investigated the impact of creatine and leucine supplementation on muscle mass loss [33]. The sample size calculation was based upon an expected >40% difference in the decline in quadriceps CSA following immobilization following nandrolone treatment (NAD) when compared to the control group. This would translate to a ~3% decline in leg muscle CSA loss in the NAD group compared to a ~5% decline in the control group. Taking into consideration drop-out rate of 10% during the experimental trial, the final number of participants who had to be included per group was 15. All participants were informed about the purpose of the study, the experimental procedures, and all its possible risks prior to providing written consent to participate. This study was approved by the Medical Ethics Committee from the Maastricht University Medical Centre+ (MUMC+), where the measurements took place. The procedures followed were in accordance with the ethical standards of the responsible institutional committee on human experimentation and in accordance with the Declaration of Helsinki of 2013.

**Table 1:** Subjects’ characteristics.

	**CON (*n* = 13)**	**NAD (*n* = 15)**
**Age (y)**	23 ± 1	22 ± 1
**Body mass (kg)**	73.1 ± 3.2	71.3 ± 2.4
**Height (m)**	1.76 ± 0.03	1.72 ± 0.02
**BMI (kg · m^-2^)**	23.5 ± 0.9	22.8 ± 0.6
**1-RM leg extension (kg)**	56 ± 4	64 ± 3
**Whole-thigh muscle CSA (mm^2^)**	14,184 ± 462	14,471 ± 386
***Quadriceps* muscle CSA (mm^2^)**	7,712 ± 324	7,805 ± 221

Data represent mean ± SEM. CON: control; NAD: Nandrolone decanoate; BMI: Body Mass Index; CSA: Cross-sectional area. 1-RM: one-repetition maximum. Baseline characteristics are not different between groups.

### Experimental outline

Eligible subjects were exposed to 7 days of muscle disuse induced by means of a full leg cast, as described previously (2). Subjects allocated to the NAD group (*n* = 15) were administered 200 mg nandrolon-17β-decanoate (Deca-Durabolin, Aspen Pharma, Dublin, Ireland) by intramuscular injection in the *gluteus maximus* muscle after the baseline measurements, prior to casting. A comparison was made with a control group. Data from the CON group have been published previously (NIH Clinical Trial Registration Number: NCT01894737) [33]. Study procedures were identical for both NAD and CON group: the immobilized leg was randomly allocated and counter-balanced between left and right. Two days prior to casting and directly after cast removal, a series of measurements were performed. Single-slice computed tomography (CT) scans of the upper leg were performed for both legs, muscle biopsies from the immobilized leg and venous blood samples were collected, and one-legged knee extension strength (one-repetition maximum [1-RM]) was assessed for both legs. All analyses were performed by investigators blinded to subject coding.

### Pretesting/ Screening

Body weight, height, blood pressure, and heart rate were measured. Subsequently, subjects’ single-leg 1-RM strength was assessed as described previously [34, 35] on a leg extension machine (Technogym, Rotterdam, The Netherlands). On the evening prior to both test days, subjects received a standardized meal containing 2.9 MJ providing 51 energy% (En%) as carbohydrate, 32 En% as fat and 17 En% as protein. Subjects were asked to maintain their habitual food intake during the study and to refrain from consuming alcohol in the 48 hours leading up to a test day. All volunteers refrained from exhaustive physical activity from 48 hours prior to the first test until the end of the study.

### Limb immobilization protocol

Two days after the first test day, subjects reported at 0800 h at the casting room at MUMC+, to have a full leg cast fitted. The application of the cast signified the first day of the 7-day immobilization period. The casting procedure has been described previously [3, 34]. In short, the circular leg cast extended from 10 cm above the ankle to ~25 cm above the patella. The knee was casted at a 30° angle of flexion to prevent subjects performing any weight bearing activities with the casted leg. Subjects were provided with crutches for proper ambulation. Throughout the immobilization period subjects were further advised to continue their normal physical activity patterns where possible, but exclude any exhaustive physical activities (such as sports activities). A physical therapist instructed all subjects how to walk the stairs safely. All subjects were instructed to perform a series of simple ankle exercises (i.e. plantar and dorsal flexion, and circular movements of the entire foot) to keep the calf muscle pump activated in the immobilized leg, thereby minimizing the risk of developing deep vein thrombosis. After 7 days of immobilization and prior to the start of the second test day, subjects visited the casting room again to have the cast removed. Thereafter, subjects were transferred by wheelchair to perform the CT scan and collect the muscle biopsy prior to any weight bearing activities.

### Muscle mass, muscle biopsies, blood sampling and strength

Subjects participated in two identical experimental test days, before and immediately after the immobilization period. Approximately 2 days prior to the immobilization period, subjects arrived at the laboratory at 0800 h in an overnight fasted state, and body weight was measured with a digital balance with an accuracy of 0.1 kg (SECA GmbH, Hamburg, Germany). Thereafter, a single-slice CT scan (Philips Brilliance 64; Philips Medical Systems, Best, The Netherlands) was performed to assess *quadriceps* muscle and upper leg muscle anatomical CSA in both legs. The scanning characteristics were as follows: 120 kV, 300 mA, rotation time of 0.75 seconds and a field of view of 500 mm. With subjects lying supine with their legs extended and feet secured, a 3 mm thick axial image was taken 15 cm proximal to the top of the patella. The precise scan position was marked with semi-permanent ink for the 7 days of immobilization to ensure accurate repeat measurements at the second test day. Muscle area of the immobilized leg was selected between -29 and 150 Hounsfield units, after which the *quadriceps* muscle was selected by manual tracing using ImageJ software (version 1.46d; National Institute of Health, Bethesda, MD, USA) [36].

Subsequently, a serum blood sample was taken from the antecubital vein by venapuncture. Thereafter, a muscle biopsy was collected from the *vastus lateralis* muscle of the leg identified as the leg to become immobilized (or the previously immobilized leg in the case of the second visit). Muscle biopsy samples were obtained from the middle region of the *vastus lateralis*, ~1–3 cm below the level that the CT scan was performed, and ~3 cm below entry through the fascia, by using the percutaneous needle biopsy technique [37]. Any visible non-muscle tissue was removed immediately, and part of the biopsy sample was embedded in Tissue-Tec (Sakura Finetek, Zoeterwoude, The Netherlands) before being frozen in liquid nitrogen-cooled isopentane, while another part was immediately frozen in liquid nitrogen. Muscle samples were subsequently stored at -80°C until further analyses. Thereafter, subjects’ single-leg 1-RM was assessed. The estimations obtained during the screening visit were used to determine 1-RM leg strength [35].

### Blood analyses

Serum was collected in SST containing tubes (SST^TM^ Tube with Silica Clot Activator), allowed to clot for at least 30 min in vertical position at room temperature and then centrifuged at 1550g and 4°C for 15 min. Aliquots of serum were frozen in liquid nitrogen and stored at –80°C until analysis.

Total nandrolone and total testosterone (T) were measured by Liquid Chromatography/Tandem Mass Spectrometry (LC/MS/MS). CVs of quality control (QC) samples were <10%. Sex hormone-binding globulin (SHBG) was measured by electrochemiluminescence immunoassay (ECLIA) (Roche Diagnostics GmbH, Mannheim, Germany) using the COBAS 8000 modular system. CVs of QC samples were <20%.

### Immunohistochemistry

Frozen muscle biopsies were cut into 5 μm thick cryosections using a cryostat at -20°C, and thaw mounted on uncoated pre-cleaned glass slides. Samples from pre- and post-immobilization were mounted together on the same glass slide. Care was taken to properly align the samples for cross-sectional fiber analyses. Muscle biopsies were stained to assess muscle fiber type distribution and CSA, as described previously [38].

Images were visualized and automatically captured at 10x magnification with a fluorescent microscope equipped with an automatic stage (IX81 motorized inverted microscope; Olympus, Hamburg, Germany) EXi Aqua CCD camera (Q Imaging, Surrey, Canada). Micromanager 1.4 software was used for image acquisition [39]. Quantitative analyses were carried out using Image J software package (version 1.45d, National Institute of Health, USA; [36]). All image recordings and analyses were performed by an investigator blinded to subject coding. Mean muscle fiber CSA was calculated for the type I and type II muscle fibers separately. Mean numbers of 115 ± 12 and 175 ± 16 fibers were analyzed in pre- and post-immobilization samples, respectively.

### Statistics

All data are expressed as mean ± SEM. Per protocol analyses were performed. Baseline values between groups were compared by means of an independent samples *t*-test. Primary outcome parameter was *quadriceps* CSA. Secondary outcome parameters were leg strength and type I and II muscle fiber size. Pre- versus post-immobilization data were analyzed using repeated-measures ANOVA with treatment (CON *vs* NAD) as between-subject factor and time (pre- *vs* post-immobilization) as within-subject factor. A *P*-value of <0.05 was used to determine statistical significance. All data were analyzed using IBM SPSS Statistics (version 25, IBM Corp., Armonk, USA).

## Results

### Subjects and dietary intake

Subjects’ characteristics are provided in Table 1. No differences were observed for age, height, weight, BMI, whole thigh, and *quadriceps* CSA and leg strength between the CON and NAD groups at baseline. Data from the CON group have been published previously (NIH Clinical Trial Registration Number: NCT01894737) [33]. Two subjects from CON withdrew prior to immobilization due to time constraints. Table 2 presents data of subjects’ habitual diet under free-living conditions and during single-leg immobilization. No differences in habitual diet were observed between groups (all measured parameters *P*>0.05). Habitual diet did not change following immobilization in either group (*P*>0.05). Habitual protein intake averaged 1.2 ± 0.1 and 1.4 ± 0.1 g·kg^-1^·day^-1^ in the CON and NAD group, respectively, with no changes following immobilization (*P*>0.05).

**Table 2:** Habitual dietary intake characteristics during free living and immobilization, with (NAD) or without (CON) nandrolone decanoate supplementation.

	**CON (*n* = 13)**		**NAD (*n* = 9)**	
	Free living	Immobilization	Free living	Immobilization
**Energy intake (MJ · day^-1^)**	7.6 ± 0.4	7.4 ± 0.6	8.8 ± 0.8	8.5 ± 0.8
**Protein (g · kg^-1^ · day^-1^)**	1.2 ± 0.1	1.1 ± 0.1	1.4 ± 0.1	1.3 ± 0.1
**Protein (En%)**	19 ± 1	18 ± 1	19 ± 1	20 ± 1
**Fat (En%)**	31 ± 2	31 ± 3	46 ± 3	49 ± 3
**Carbohydrate (En%)**	54 ± 8	47 ± 3	34 ± 4	29 ± 3

Data represent mean ± SEM. CON: control; NAD: Nandrolone decanoate. En%: Energy %. Data were analyzed using repeated-measures ANOVA. No differences between leucine and control were found over time.

### Muscle mass and leg strength

**Fig 2:**
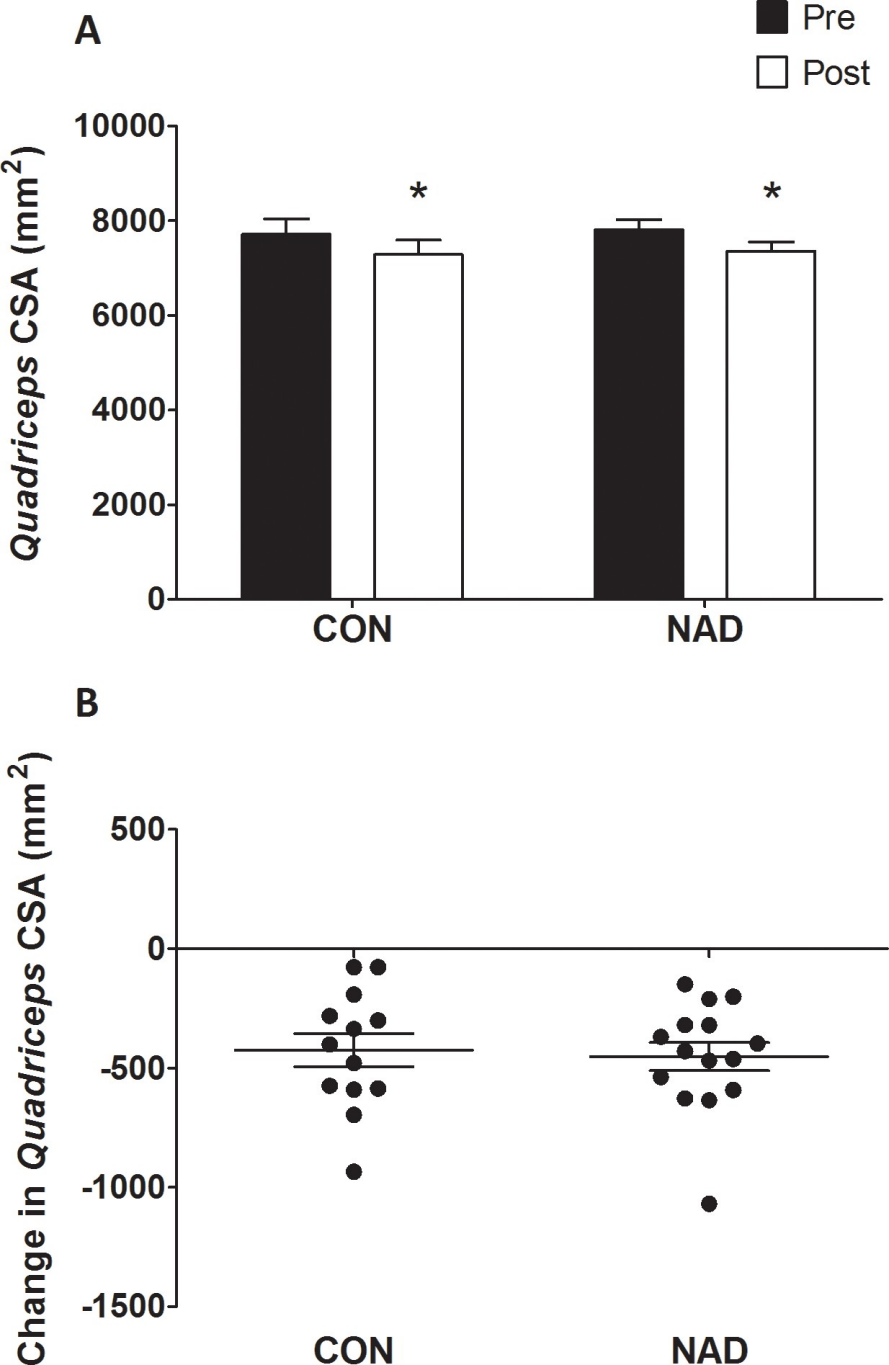
2A: Cross-sectional area (CSA) of *quadriceps* muscle in the CON and NAD group, before and after 7 days of leg immobilization, as measured by single-slice CT scan. 2B: Individual changes in *quadriceps* muscle CSA following 7 days of one-legged knee immobilization. Data were analyzed using repeated-measures ANOVA. Data are expressed as mean±SEM. Immobilization resulted in a significant decline in *quadriceps* muscle CSA in both groups (* *P*<0.05), with no differences between groups.

**Fig 3:**
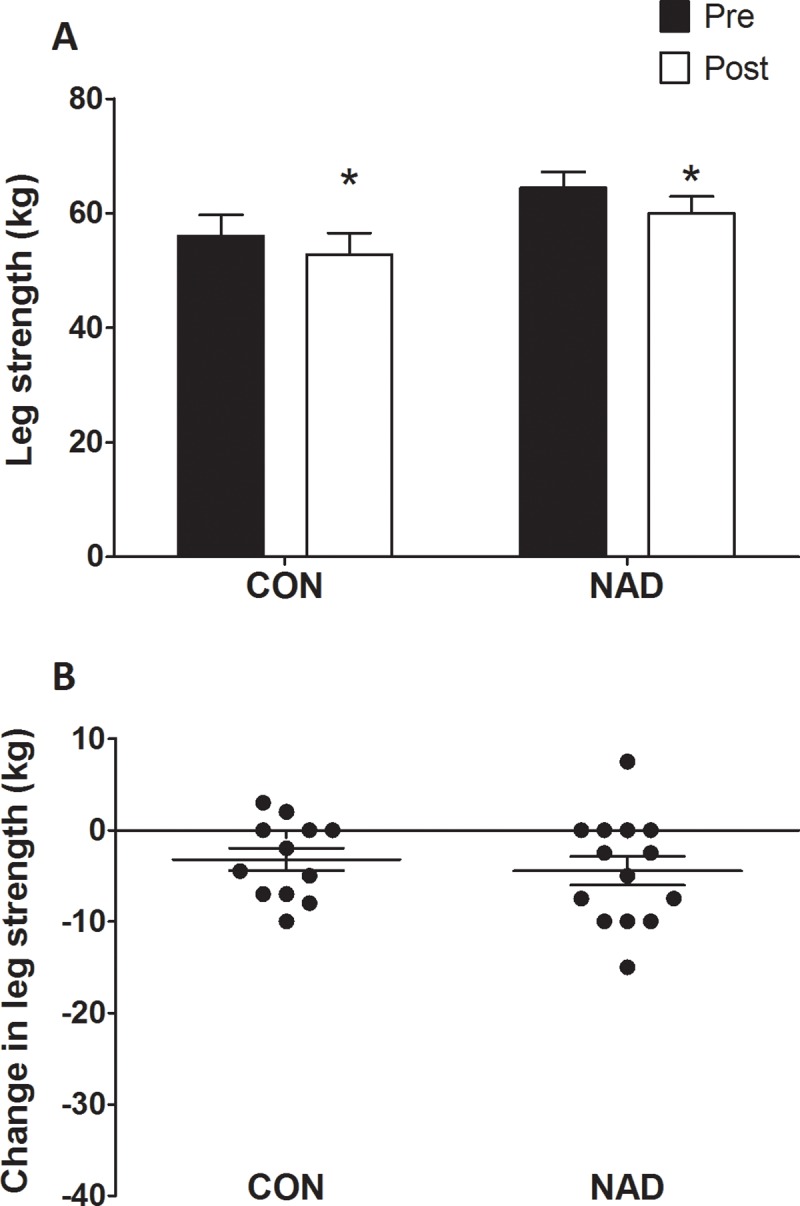
3A: Leg muscle strength as measured by one-repetition maximum (1-RM), in the CON and NAD group before and after 7 days of one-legged knee immobilization. 3B: Individual changes in 1-RM leg muscle strength following 7 days of one-legged knee immobilization. Data were analyzed using repeated-measures ANOVA. Data are expressed as mean±SEM. Immobilization resulted in a significant decline in muscle strength in both groups (* *P*<0.05), with no differences between groups.

*Quadriceps* muscle CSAs are displayed in Fig 2A. At baseline, no differences were observed in *quadriceps* muscle CSA between groups (*P* = 0.59). Seven days of immobilization caused a significant reduction in *quadriceps* muscle CSA (from 7,712 ± 324 mm^2^ to 7,287 ± 305 mm^2^ and from 7,805 ± 221 mm^2^ to 7,352 ± 202 mm^2^ in the CON and NAD group, respectively (time *P*<0.01). No differences were observed in muscle loss between CON (-5.5 ± 0.8%) and NAD (-5.8 ± 0.7%; *P*>0.05). Fig 2B shows the individual changes in *quadriceps* CSA following 7 days of one-legged knee immobilization. Leg strength data are presented in Fig 3A. Maximal leg strength decreased following immobilization from 56 ± 4 kg to 53 ± 4 kg (-5.6 ± 2.2%) and from 64 ± 3 kg to 60 ± 3 kg (-6.9 ± 2.6%) in the CON and NAD group, respectively (main effect of time, *P*<0.05), with no differences between groups (time × treatment interaction, *P* = 0.55). Fig 3B shows the individual changes in maximal leg strength following 7 days of one-legged knee immobilization.

### Blood analyses

In general serum nandrolone concentrations are not detectable below levels of 0.01 nmol/L in healthy men [40]. At baseline and in the CON group also during immobilization, we have not measured levels above this detection limit. In the NAD group, after injection, serum nandrolone concentrations increased to 13.8 ± 1.0 nmol/L during the immobilization period. As expected, total testosterone concentrations decreased in the NAD group (from 22.2 ± 7.4 to 1.5 ± 1.7 nmol/L) and did not change in the CON group (20.8 ± 5.1 and 20.6 ± 4.8 nmol/L), pre- and post-immobilization, respectively (time × treatment interaction, *P*<0.001). The concentration of SHBG did not differ at baseline (32 ± 2 and 32 ± 3 nmol/L; *P* = 0.927) and did not change following immobilization (31 ± 2 and 31 ± 3 nmol/L for CON and NAD, respectively (*P*>0.05).

### Muscle fiber characteristics

Muscle fiber characteristics are displayed in Table 3. At baseline, no significant differences between groups were observed for any of the variables. No changes in type I and type II muscle fiber CSA or distribution were observed following immobilization (*P*>0.05), and no differences were observed between groups (*P*>0.05).

**Table 3:** Muscle fiber characteristics of healthy young men before (pre) and after (post) 7 days of leg immobilization, with (NAD) or without (CON) nandrolone decanoate supplementation.

		CON (*n* = 13)		NAD (*n* = 15)	
	Fiber type	Pre	Post	Pre	Post
**Muscle fiber CSA (μm^2^) (μm^2^)**	I	6034 ± 501	6620 ± 508	6014 ± 283	6188 ± 253
	II*	7202 ± 640	7540 ± 587	6587 ± 512	6594 ± 311
**% Fiber**	I	38 ± 4	33 ± 3	40 ± 3	40 ± 4
	II*	62 ± 4	67 ± 3	60 ± 3	60 ± 4

Data represent mean ± SEM. Abbreviations: CON: control; NAD: Nandrolone decanoate; CSA: Cross-sectional area. No interaction or time effect was found in any of the variables.

* Muscle fiber CSA and fiber type distribution (in %) were different between type I and type II muscle fibers on all time points.

## Discussion

In the present study, we demonstrated that a single intramuscular injection of nandrolone decanoate (200 mg) does not attenuate the decline in muscle mass and strength during 7 days of subsequent leg immobilization in healthy male adults.

A prolonged period of skeletal muscle disuse or short, successive episodes of muscle disuse can strongly reduce skeletal muscle mass and strength and cause numerous negative health outcomes [41–45]. It is now believed that short, successive periods of muscle disuse due to injury or disease may be largely responsible for the progressive loss of skeletal muscle mass observed throughout the lifespan [1, 4]. In the present study, we observed that 7 days of leg immobilization causes substantial loss of muscle mass (see Fig 2A). The ~6% decline in *quadriceps* cross-sectional area was accompanied by a similar (6–7%) decline in leg muscle strength. These observations are in line with recent data from our group [3, 12] as well as others [46] reporting a 2–8 and 8–23% decline in muscle mass and strength, respectively, following 1–2 weeks of disuse in both young and older individuals. These data are of important clinical relevance as hospitalization following acute illness or injury is generally accompanied by an average hospital stay of 6–7 days [2]. The loss of muscle mass and strength during such short (successive) periods of muscle disuse impairs functional capacity, increases the risk of developing chronic metabolic disease, and hinders the subsequent rehabilitation upon discharge [47]. Clearly, effective interventional strategies are needed to prevent or attenuate muscle mass and strength loss during short periods of muscle disuse due to injury, disease, and/or surgery.

Administration of the anabolic androgenic steroid nandrolone has previously been shown to increase fat free mass, muscle cross-sectional area, and/or strength in several pathological conditions when applied for 1–6 months with doses ranging between 200–2400 mg [20–30]. Furthermore, nandrolone decanoate administration in healthy men has been shown to increase fat-free mass and muscle size and strength, especially when combined with resistance type exercise training [48]. In the present study, we administered a single intramuscular 200 mg dose of nandrolone decanoate immediately prior to immobilization. The injection was well received and no adverse events were reported following nandrolone decanoate administration during the subsequent immobilization period. Serum nandrolone concentrations significantly increased in the NAD group up to 6.0 ng/mL which agrees well with the peak of 5.2 ng/mL after an intramuscular injection of 150 mg in healthy men in a pharmacokinetic evaluation study of different doses of nandrolone decanoate [14]. In addition, our increase in nandrolone concentrations and decrease in total testosterone concentrations from 6.4 to 0.4 ng/mL are approximately double the concentrations that were found a week after subcutaneous injection of half of our dose, i.e. 100 mg nandrolone decanoate in another pharmacokinetic-pharmacodynamic study [49]. In short, our intervention was quite effective in increasing nandrolone decanoate concentrations and in line with previous observations. Despite the substantial increase in serum nandrolone concentrations, no differences were observed in the loss of muscle mass and/or strength between the NAD and CON group following single leg immobilization.

Although *quadriceps* CSA declined by 6±1% in response to immobilization in both groups, such changes were not yet evident on the muscle fiber level. The lack of measurable declines in type I or type II muscle fiber CSA are in line with previous publications investigating changes in muscle fiber characteristics following a relative short (~7 days) period of physical inactivity [12, 34], and is attributed to the large variance in fiber size between fibers.

In the present study, we chose to provide nandrolone decanoate on the day of immobilization (as opposed to several days or weeks prior to bed rest or immobilization) as it presents a practical and clinically relevant strategy for more clinically compromised patients being hospitalized following both acute injury as well as scheduled surgery. A previous study on nandrolone decanoate pharmacokinetics has shown serum nandrolone levels to peak 72 hours after intramuscular injection, with the half-life of 7–12 days [14]. In agreement, circulating nandrolone levels were elevated substantially throughout the 7 days of disuse. Nonetheless, we failed to detect any impact of nandrolone decanoate administration on muscle mass or strength loss during single-leg limb immobilization. We can only speculate on the efficacy of higher doses of nandrolone decanoate or nandrolone decanoate administration in the weeks prior to the onset of disuse. The latter would be of interest but only applicable for those patients with elective, planned surgery. Clearly, the proposed benefits of nandrolone decanoate on preventing or attenuating muscle mass and strength loss during disuse are not as evident as anticipated. The present study shows that nandrolone decanoate administration does not represent an effective adjuvant pharmaceutical strategy to prevent or attenuate muscle disuse atrophy.

Prior work from our group has shown that preservation of muscle mass during disuse is possible by applying exercise mimetics such as neuromuscular electrical stimulation. Daily application of neuromuscular electrical stimulation was shown to preserve muscle mass in a leg immobilization model in healthy volunteers [34] as well as in comatose patients in an intensive care unit setting [50]. It could be speculated that local muscle contraction is required to allow preservation of skeletal muscle mass and function. It could be speculated that nandrolone decanoate could support preservation of muscle mass and/or strength when applied in combination with exercise (mimetics). The latter is supported by the observation that nandrolone decanoate administration has been shown to be (more) potent to support muscle hypertrophy when combined with (prolonged) resistance type exercise training [48]. Adjuvant pharmacological support with nandrolone decanoate may be (more) supportive when combined with exercise (mimetics) during disuse or rather to augment muscle mass and strength gains during the subsequent rehabilitation process [24, 27, 28, 32]. Therefore, future work should evaluate the potential benefits of NAD treatment when combined with exercise or exercise mimetics applied during hospitalization and/or subsequent rehabilitation following a period of disuse. Furthermore, dose response studies should evaluate the impact of timing and dosing of nandrolone decanoate under such conditions.

## Conclusions

Administration of a single bolus of nandrolone decanoate (200 mg) prior to a short period of disuse does not attenuate muscle mass or strength loss. This is the first study to report no preservation of skeletal muscle mass and strength following nandrolone decanoate administration during a short period of leg immobilization *in vivo* in humans.

## Supporting information

S1 Trend Checklist (PDF)

S1 File METC protocol NL44547.068.13. (PDF)

S2 File METC protocol NL50679.068.15. (PDF)
