# Supplementary material for: Nandrolone decanoate administration does not attenuate muscle atrophy during a short period of disuse
Source: PLoS One. 2019 Jan 28;14(1):e0210823. doi: 10.1371/journal.pone.0210823 (PMC6349315; doi:10.1371/journal.pone.0210823)
Supplement: S1 File — (PDF) [file pone.0210823.s002.pdf]

The effect of Creatine Supplementation on Muscle Loss During Immobilisation

The Effect of Creatine Supplementation on Muscle Loss During Immobilisation

**Protocol ID**	**NL44547.068.13 (METC 13-3-023)**
**Short title**	**Creatine and disuse atrophy**
**Version**	**2**
**Date**	**21-6-2013**
**Coordinating investigator/project leader**	Prof. Luc van Loon, PhD<br>Human Movement Sciences<br>MUMC+<br>l.vanloon@maastrichtuniversity.nl<br>+31-43-3881397
**Principal investigators**	Dr. Lex Verdijk, PhD<br>Human Movement Sciences<br>MUMC+<br>lex.verdijk@maastrichtuniversity.nl<br>+31-43-3881318<br><br>Dr. Ben Wall, PhD<br>Human Movement Sciences<br>MUMC+<br>Benjamin.wall@maastrichtuniversity.nl<br>+31-43-3882215<br><br>Mr. Tim Snijders, MSc<br>Human Movement Sciences<br>MUMC+<br>Tim.snijders@maastrichtuniversity.nl<br>+31-43-3881390
**Sponsor**	Nutrim School for Nutrition, Toxicology and Metabolism, UM
**Responsible physician**	Bart Groen, MD<br>Human Movement Sciences<br>MUMC+<br>bart.groen@maastrichtuniversity.nl<br>+31-43-3881390
**Independent physician(s)**	Martijn Poeze, MD, PhD<br>Department of Surgery, division of Traumatology<br>MUMC+<br>m.poeze@mumc.nl
**Laboratory sites**	UM (Human Movement Sciences)

PROTOCOL SIGNATURE SHEET

Name	Signature	Date
**Coordinating Investigator / Project Leader:**<br><br>Prof. L.J.C. van Loon<br>(Professor, Human Movement Sciences, MUMC+)		
**Principal investigators:**<br><br>Dr. Lex Verdijk<br>(Assistant professor, Human Movement Sciences, MUMC+)<br><br>Dr. Ben Wall<br>(Post-doc, Human Movement Sciences, MUMC+)<br><br>Tim Snijders, MSc<br>(PhD student, Human Movement Sciences, MUMC+)		

TABLE OF CONTENTS

1. INTRODUCTION AND RATIONALE 10
2. OBJECTIVES 12
3. STUDY DESIGN 13
   - 3.1 Screening 14
   - 3.2 Diet and activity prior to testing 14
   - 3.3 Pre creatine loading test day 15
   - 3.4 Pre and post immobilisation test days 15
   - 3.5 Immobilisation protocol 16
   - 3.6 Recovery Measurement Day 17
4. STUDY POPULATION 18
   - 4.1 Inclusion criteria 18
   - 4.2 Exclusion criteria 18
   - 4.3 Sample size calculation 19
5. TREATMENT OF SUBJECTS 20
   - 5.1 Investigational treatments 20
     - 5.1.1 Seven days of immobilisation via a full leg cast 20
     - 5.1.2 Creatine supplementation 21
   - 5.2 Use of co-intervention 21
6. INVESTIGATIONAL PRODUCT 22
   - 6.1 Name and description of investigational product 22
   - 6.2 Summary of Findings from Non-Clinical Studies 22
   - 6.3 Summary of known and potential risks and benefits 22
   - 6.4 Description and justification of route of administration and dosage 23
   - 6.5 Dosages, dosage modifications and method of administration 23
   - 6.6 Preparation and labelling 24
7. METHODS 26
   - 7.1 Study parameters/endpoints 26
     - 7.1.1 Main study parameter/endpoint 26
     - 7.1.2 Secondary study parameters/endpoints 26
     - 7.1.3 Plasma and serum samples 26
     - 7.1.4 Muscle biopsies 26
     - 7.1.5 Other study parameters 27
   - 7.2 Randomization, blinding and treatment allocation 27
   - 7.3 Study procedures 27
     - 7.3.1 Screening & pre-testing 27
     - 7.3.2 Diet and activity prior to testing 27
     - 7.3.3 Experimental test days (3x) 28
     - 7.3.4 Analyses 28
   - 7.4 Withdrawal of individual subjects 29
   - 7.5 Replacement of individual subjects after withdrawal 29
   - 7.6 Follow-up of subjects withdrawn from treatment 29

7.7 Premature termination of the study....29
8. SAFETY REPORTING....30
Section 10 WMO event....30
8.1 Adverse and serious adverse events....30
8.2 Follow-up of adverse events....31
9. STATISTICAL ANALYSIS....32
10. ETHICAL CONSIDERATIONS....33
10.1 Regulation statement....33
10.2 Recruitment and consent....33
10.3 Benefits and risks assessment, group relatedness....33
10.4 Compensation for injury....35
10.5 Incentives (if applicable)....35
11. ADMINISTRATIVE ASPECTS AND PUBLICATION....37
11.1 Handling and storage of data and documents....37
11.2 Amendments....37
11.3 Annual progress report....37
11.4 End of study report....37
11.1 Public disclosure and publication policy....38
12. STRUCTURED RISK ANALYSIS....39
12.1 Potential issues of concern....39
12.1.1 Muscle cramps....39
12.1.2 Gastrointestinal complaints....39
12.1.3 Kidney problems....39
12.1.4 Water retention....39
12.1.5 Dosage....40
12.2 Synthesis....40
13. REFERENCES....41

LIST OF ABBREVIATIONS AND RELEVANT DEFINITIONS

**ABR**	ABR form, General Assessment and Registration form, is the application form that is required for submission to the accredited Ethics Committee (In Dutch, ABR = Algemene Beoordeling en Registratie)
**AE**	Adverse Event
**AR**	Adverse Reaction
**BMI**	Body Mass Index
**CA**	Competent Authority
**CCMO**	Central Committee on Research Involving Human Subjects; in Dutch: Centrale Commissie Mensgebonden Onderzoek
**CR**	Creatine treatment group
**CSA**	Cross-sectional area
**CT**	Computed Tomography
**CV**	Curriculum Vitae
**DSMB**	Data Safety Monitoring Board
**DVT**	Deep Vein Thrombosis
**EU**	European Union
**EudraCT**	European drug regulatory affairs Clinical Trials
**FDR**	False Discovery Rate
**GCP**	Good Clinical Practice
**HMB**	β-hydroxy-β-methylbutyrate
**IB**	Investigator's Brochure
**IC**	Informed Consent
**IGF-1**	Insulin-like Growth Factor 1
**IMP**	Investigational Medicinal Product
**IMPD**	Investigational Medicinal Product Dossier
**METC**	Medical research ethics committee (MREC); in Dutch: medisch ethische toetsing commissie (METC)
**RMA**	Robust Multi-array Average
**(S)AE**	(Serious) Adverse Event
**SPC**	Summary of Product Characteristics (in Dutch: officiële productinfomatie IB1-tekst)
**Sponsor**	The sponsor is the party that commissions the organisation or performance of the research, for example a pharmaceutical company, academic hospital, scientific organisation or investigator. A party that

provides funding for a study but does not commission it is not regarded as the sponsor, but referred to as a subsidising party.

SUSAR Suspected Unexpected Serious Adverse Reaction

PLA Placebo treatment group

Wbp Personal Data Protection Act (in Dutch: Wet Bescherming Persoonsgevens)

WMO Medical Research Involving Human Subjects Act (in Dutch: Wet Medisch-wetenschappelijk Onderzoek met Mensen

1-RM One-repetition maximum

SUMMARY

Rationale: Muscle loss can occur for several reasons, such as inactivity because of illness or injury, illnesses themselves or simply old age. Limiting muscle loss is of profound importance, because loss of muscle mass can have a strong impact on quality of life. One possible strategy is to supplement with the compound creatine. Creatine may reduce loss of skeletal muscle in catabolic situations. We intend to investigate creatine's ability to attenuate muscle loss, both from a physiological and mechanistic standpoint. We will use short-term immobilisation of the knee as a model to induce muscular atrophy.

Objective: To determine the effect of creatine supplementation on muscle mass loss during short-term immobilisation in healthy, young men.

Study design: Randomized, double-blind placebo-controlled parallel study.

Study population: 30 young healthy volunteers (age 18-35 years old, 18.5 < BMI < 30 kg/m^2).

Intervention: Seven days of one-legged knee immobilisation in young subjects with creatine or placebo supplementation. The supplementation consists of five days of creatine (or placebo) loading (20 g/d) prior to the seven days of immobilisation. After the initial creatine loading phase, daily creatine (or placebo) intake is reduced to 5 grams per day, which is maintained for the duration of the study. A randomly selected leg is immobilized for seven days. Muscle mass and strength will be determined and muscle biopsies will be collected before and immediately after immobilisation. A week after the cast is removed we will assess any differences in recovery between intervention groups.

Main study parameters/endpoints:

Primary: quadriceps muscle cross sectional area (CSA) by CT scan.

Secondary: Leg muscle strength (1RM), type I and II muscle fiber size and satellite cell content, muscle transcriptomics, muscle and blood metabolomics, full upper leg muscle CSA.

Nature and extent of the burden and risks associated with participation, benefit and group relatedness:

The risks involved in participating in this experiment are minimal. Muscle biopsies will be taken through a small (5 mm) incision, following local anesthetics of the skin and muscle fascia, and will heal completely. Muscle biopsies will only be obtained by an experienced physician, minimizing the risk for infection and local hematoma. Seven days of limb immobilisation via a full leg cast will impair subject's mobility for this period and may increase the risk of deep vein thrombosis. To minimize any risk of injury, subjects will not be allowed to drive a vehicle or ride a bicycle and will have daily contact with the investigators. The seven day immobilisation period will lead to a loss of muscle mass and strength. However, the expected loss of muscle mass and strength following immobilisation will be rapidly (<4

weeks) regained due to the inclusion of only healthy volunteers. The CT scans used to measure changes in muscle CSA will expose study participants to additional radiation (0.159 mSV in total). Side effects of creatine supplementation have been reported: cramps, gastrointestinal problems and water retention (weight gain). Gastrointestinal problems and cramps are rare and may be based on anecdotal evidence or improper creatine usage. Water retention is temporary and is only present while using supplemental creatine.

1. INTRODUCTION AND RATIONALE

Muscle loss can occur for several reasons, such as inactivity because of illness or (sports) injuries, illnesses themselves or simply old age. A decrease in muscle mass can have a profound impact on sports performance, functional performance, and quality of life, as it can lead to decreased strength and power, but also insulin resistance, lower basal metabolic rate and obesity [1-6]. One way to induce muscle loss and study its effects is immobilisation. Previous studies have shown that immobilisation of the knee can rapidly induce muscle atrophy [7-13]. Previous studies performed at MUMC+ have shown that immobilisation can cause a reduction of 3.5% in quadriceps muscle cross-sectional area in as little as five days in young men (MEC 11-3-073, manuscript in preparation). Correspondingly, muscle strength is substantially reduced in these subjects (i.e. ~10%). As such, immobilisation is a suitable model to induce loss of muscle mass and strength and, therefore, can be used to test nutritional strategies to attenuate muscle loss during situations of muscle disuse and/or injury. Several nutrients have shown promise regarding the protection of muscle mass in catabolic situations, one of which is creatine [14]. Creatine is a naturally occurring compound and plays an important role in muscular energy metabolism. It is synthesised by the human body, although approximately half of the body's stores of creatine originate from food [15].

Creatine has been well established as an anabolic compound and a wide range of studies have shown that creatine is capable of enhancing the training effect of resistance exercise, improving body composition and increasing muscular strength and endurance (for reviews see [15-17]). In the short term, creatine can improve performance by increasing the store of creatine phosphate in the muscle, which leads to a higher capacity to perform resistance exercise. By increasing the work capacity creatine can increase the muscle growth stimulus over time. Several studies have also shown that creatine is capable of stimulating muscle growth independently of training stimuli and, importantly, may be capable of attenuating muscle loss in certain situations [13, 14, 18-20].

A previous study has shown that creatine strongly attenuates muscle loss during immobilisation of the upper extremity [14]. In fact, this study shows a slight increase in lean muscle mass of the immobilized arm in the creatine group. This increase is likely because of water retention, a known effect of creatine. Muscle strength and endurance slightly decreased in the creatine group after immobilisation, but significantly less strength was lost in the creatine group than the control group. The question remains however whether creatine has the same protective effects on leg muscles, which are larger and usually more active during day-to-day living than arm muscles because of their key role in weight bearing

activities. Moreover, assessing potential protective effects of creatine supplementation on lower limb muscle mass maintenance during disuse has more clinical relevance, as the legs are the primary site of muscle loss during illness and (sports) injuries [21]. An earlier study, using leg immobilisation, has shown that when creatine is given during and after immobilisation, post-immobilisation recovery is accelerated [13]. However, the effect of creatine on lower extremity muscle loss *during* immobilisation remains to be established.

While creatine is a well-established and commonly used supplement, it is still not fully understood what the mechanism of action behind creatine is. One potential mechanism is its ability to activate satellite cells. In the study by Hespel et al. [13] creatine was found to significantly alter myf6 and myogenin gene expression, two myogenic regulatory transcription factors that have been shown to be involved in myogenesis and muscle growth. Other studies have reported similar effects of creatine on myogenic regulatory transcription factors and satellite cells [19, 22]. Another potential mechanism is the activation and potentiation of anabolic signalling pathways. Several studies have shown that creatine improves IGF-1 signalling for example, which is an important anabolic signalling pathway [23-25].

The present study is a proof-of-principle study to determine the potential of creatine to attenuate muscle loss during immobilisation, as well as the underlying mechanisms. The findings may be directly translated to healthy young individuals (e.g. athletes) that suffer from injury for which immobilisation is induced. Indirectly, insight from this study may reveal potential targets for further study in clinical conditions, such as in more compromised elderly and/or hospitalized patients. In order to study the underlying mechanisms of disuse muscle atrophy, and the potential beneficial impact of creatine hereon, we intend to immobilize the knee of 30 young subjects for seven days. Subjects will be supplemented with creatine (n=15) or a placebo (n=15) to determine its efficacy for attenuation of muscle mass and strength loss during disuse. We also want to investigate whether creatine influences the early recovery following immobilisation. In addition to determining the physiological/functional effects of immobilisation and creatine supplementation (i.e. muscle mass and function), we intend to use metabolomics and whole genome transcriptomics to determine the underlying mechanisms of disuse atrophy on the one hand, and creatine’s mechanism of action on (potentially) attenuating muscle loss on the other hand. Using transcriptomics we can determine which genes are involved in muscle loss and which genes are activated or inactivated by creatine supplementation. Metabolomics can for example shed light on changes in the levels of amino acids and metabolites of amino acids, such as HMB (β-hydroxy-β-methylbutyrate), which may have a potential role in muscle atrophy.

2. OBJECTIVES

The primary aim of this study is to determine the effect of creatine supplementation on muscle mass loss during short-term immobilisation in healthy, young people. In addition, we aim to study the underlying mechanisms of creatine and disuse muscular atrophy. The latter is more explorative in nature.

We intend to test the following hypotheses:

1. Creatine supplementation during immobilisation of the knee reduces muscle loss in the upper leg
2. Creatine supplementation during and after immobilisation improves the recovery of lost muscle CSA

In addition, we intend to answer the following research questions:

1. Which genes are up- or down-regulated in skeletal muscle tissue by creatine supplementation?
2. Which genes are up- or down-regulated in skeletal muscle tissue by immobilisation?
3. What is the effect of creatine supplementation during immobilisation on muscle fiber size and satellite cell content?
4. What is the effect of creatine supplementation during immobilisation on the muscle metabolome?

3. STUDY DESIGN

The present study will use a randomised, double-blind, placebo-controlled parallel-arm study design with two groups. All volunteers (n=30) will be subjected to 7 days of one legged knee immobilisation by means of a full leg cast, either with (n=15, creatine group) or without (n=15, control group) creatine monohydrate supplementation. The creatine group will be loaded for 5 days prior to immobilisation by providing 20 g of creatine per day. This will ensure that muscular creatine stores are at maximal capacity before the leg is immobilised [13, 26, 27]. After the loading phase creatine monohydrate dosage will be reduced to a maintenance dose of 5 g per day, which will be taken during immobilisation and during the recovery week after the immobilisation.

Previous studies performed within our research group have shown that five days of immobilisation can induce a loss in muscle CSA of approximately 3.5% in young individuals (i.e. ~ 0.7% per day). Previous studies have shown that muscle loss during immobilisation is linear in the first two weeks. This has been demonstrated by our group (MEC 11-3-073 and 09-3-011) and by studies performed at other universities as was recently reviewed [21]. Consequently, we expect the decrease in CSA to be approximately 5% after the seven days of immobilisation in our study. We have slightly increased the duration of immobilisation from 5 to 7 days to limit the number of subjects needed to show a benefit of creatine supplementation (also see 4.3: Sample size calculation; with only 5 days of immobilisation, the number of subjects needed would be almost doubled). Blood and muscle samples are taken before creatine loading, before immobilisation, after immobilisation and one week after cast removal. In addition, CT scans will be performed before and after immobilisation, and after 1 week recovery to determine changes in muscle size. Blood and muscle samples will be used for metabolome analysis. Additional analyses (e.g. vitamin D, glucose, insulin, IGF-1, testosterone, cortisol) will be measured in the blood samples when appropriate. Changes in gene expression in the muscle samples will be determined using microarrays. 1-RM tests will be performed before and after immobilisation in order to measure changes in muscle strength. Figure 1 presents an overview of the study outline.

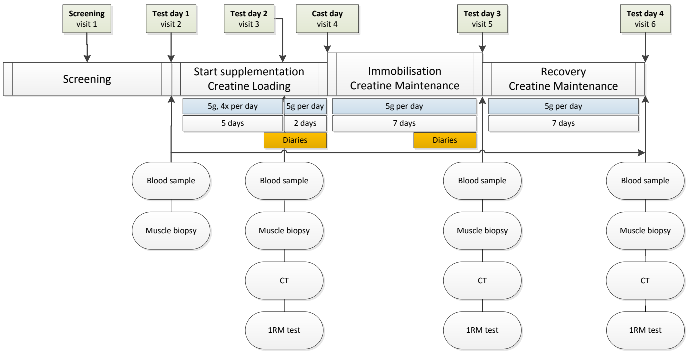

Figure 1: Overview of the Study Design

3.1 Screening

Prior to the study, volunteers' suitability to participate will be assessed in a single screening session. After explaining all procedures, written informed consent will be obtained from subjects willing to participate. We will determine body weight, height and leg volume using basic anthropometry. Subjects will be asked to fill in a medical questionnaire enquiring about their general health, medical history, use of medication and sports activities. This questionnaire will be carefully assessed by the responsible physician before subjects are allowed entry into the study. Subjects with known impaired renal function will be excluded from participation.

Strength will be estimated on the screening day using a sub-maximal strength test. This estimate is later used for a 1-RM strength test where the actual strength is determined. During the 1-RM test, subjects will be asked to take place on a Leg Extension machine. By raising the weight that is lifted after every set, eventually the weight that can be lifted only once is determined; this is the subject's One Repetition Maximum (1-RM). The 1-RM test will be performed with both legs separately to determine single-legged 1-RM.

Volunteers will be instructed on the proper use of crutches which will be necessary for the immobilisation period. In the event of any unexpected medical findings during the screening, subjects will always be notified. If a subject does not want to receive this notification he cannot participate in the study. Our policy behind co-incidental findings is that we will inform the subjects' general practitioner about the results that were found. If subjects do not want this, they cannot participate in the study. Following screening and acceptance for the study, volunteers will be given a unique randomization number, which automatically allocates each individual to one of the two experimental groups (placebo or creatine).

3.2 Diet and activity prior to testing

All subjects will consume a standardized dinner the evening before the test day. This standardized dinner is an 'Aviko maaltijdpannetje' and will be purchased at a regular supermarket in Maastricht. The expiration date from the manufacturer will be checked. The meals will be stored in an appropriate freezer of the 'dietary-kitchen' at the department of Human Biology. The subjects will receive the meal after the screening visit in a thermal bag. The precise composition and preparation methods are described in Appendix D4.1. The subjects will be instructed to store the meal in a freezer until preparation and to prepare the meal themselves according to the instructions on the label. Subjects will be instructed to refrain from any sort of heavy physical exercise over the course of the study and not deviate

from their normal eating habits. In addition, subjects will be asked to record their food intake and physical activity for 3 days before the first data collection test day in specific diaries.

3.3 Pre creatine loading test day

The first test day will be carried out one day before the start of creatine (or placebo) supplementation. These measurements are to determine the baseline before creatine supplementation begins. During this session we will take a blood sample and a muscle biopsy.

The muscle biopsy will be obtained from the middle region of the *m. vastus lateralis* (15 cm above the patella and approximately 2 cm away from the fascia) by the percutaneous needle biopsy technique [28]. Muscle biopsies will be carefully freed from any visible fat and blood and part of the sample will be rapidly frozen in liquid nitrogen cooled isopentane, embedded in Tissue-Tek (Sakura Finetek Europe BV, The Netherlands) with the remaining part being frozen immediately in liquid nitrogen. Muscle biopsies will be stored at –80 ºC for subsequent analysis. The leg chosen to obtain the muscle biopsy from and, consequently, the one to be immobilized, will be randomized. A single blood draw will also be obtained by venipuncture. Subjects will receive a breakfast after samples have been taken.

3.4 Pre and post immobilisation test days

Before and after immobilisation there are two test days. These sessions last approximately 3 hours. The first test day will be carried out two days before immobilisation and marks the end of the loading phase. These measurements are to determine any effects of creatine itself on the muscle before immobilisation. During this session we will do a CT scan, take a blood sample and perform a muscle biopsy. Subjects will arrive at the laboratory at 08.00 am in the fasted state having not consumed anything (except water) since the controlled meal (described above, section 3.2) at ~08.00 pm the previous evening. We will also measure leg volume by anthropometry on the day the cast is removed (post immobilisation).

First, a single slice CT scan (IDT 8000; Philips Medical Systems, Best, Netherlands) at 15 cm above the base of the patella will be performed in the Academic hospital of Maastricht (department of radiology) to determine (anatomical) cross sectional area of the *quadriceps femoris* muscle of both legs. While the participants will be supine with their legs extended and their feet secured, a 3-mm thick axial image will be taken. The scanning characteristics will be as follows: 120kV, 300mA, rotation time of 0.75 sec, and a field of view of 500 mm. The exact scanning position will be measured and marked for replication.

Thereafter, a second muscle biopsy will be obtained from the leg to be immobilized as described above (3.3). Also, a blood sample will be obtained by venipuncture. Subsequently, 1-RM strength will be determined. After warming up, the load will be set at 95% of the estimated 1-RM (obtained during screening), and increased after each successful lift until failure [29]. 3 min rest periods will be allowed between attempts. A repetition is valid if the subject uses proper form and is able to complete the entire lift in a controlled manner without assistance. Muscle biopsies are taken in the basal state before the 1RM test to ensure the maximal effort does not interfere with gene expression. This has been done before without problems (e.g. MEC 09-3-080).

After completion of the measurements, subjects will receive a breakfast that consist of whole-wheat bread, 48+ cheese and sweet spreads such as chocolate paste.

3.5 Immobilisation protocol

Two days following the pre immobilisation test day (section 3.4), a 7 day period of immobilisation will commence. Volunteers will arrive the first day at the laboratory at 08.00 am by public transport. The muscle biopsy wound will be inspected carefully by the responsible physician and with his approval the leg will be immobilized with a cast: from 10 cm above the ankle to half way the upper leg (30 degree flexion of the knee-joint; see Fig 2). Next, the subject will be instructed again on how to use crutches and to not bear weight on the casted limb, and to minimize muscle contractions of the upper leg. A previous immobilisation study (MEC 09-3-011) has employed the same full leg cast protocol for a period of two weeks as a means of immobilizing the leg. Thus, the present study will pose much less of a burden given the shorter duration of immobilisation (7 days).

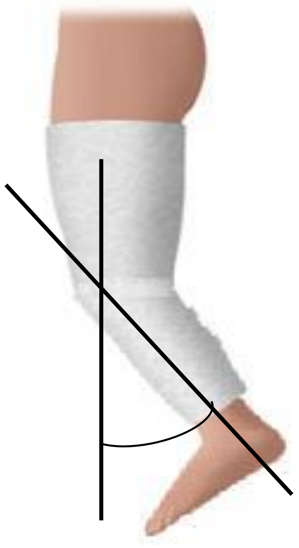

Figure 2: Representation of a full leg cast at a knee angle of 30 degrees of flexion

Following the fitting of the cast, transport home from the university will be arranged either by car or taxi. During the seven days of immobilisation, all subjects will be checked upon daily by telephone by a member of the research team. These check-ups will serve to answer any questions/concerns from the participant and to check compliance with the protocol.

The day after the seven days of immobilisation (i.e. day 15 of the test period) volunteers will be provided with transport (car or taxi) to arrive at the laboratory at 8.00 am. First, the cast will be removed and then the volunteer will not be allowed to place any weight on the leg. Instead, the subject will be transported in a wheelchair to the CT scanner where a single slice CT scan will be performed as described above. Thereafter, subjects will be transported by wheelchair to the laboratory where a third muscle biopsy and blood sample will be collected as described above. Thus, the biopsy will be collected while the subject is still in the immobilized state (i.e. before they have performed any weight bearing muscle contractions). Leg volume will be measured after the muscle biopsy has been taken. Following the muscle biopsy and blood sample, 1RM strength testing will be performed as described above.

3.6 Recovery Measurement Day

Seven days after cast removal, subjects will be required to attend the laboratory in a fasted state on one more occasion. This visit serves to determine whether creatine influences the early recovery of the subjects. On this day we will perform a CT scan and 1-RM measurements in exactly the same manner as described above. We will take a fourth blood sample and we will also take a fourth muscle biopsy to assess tissue plasticity and recovery. Like other test days, we will start with the CT-scan, followed by collection of the muscle biopsy and blood sample and ending with the 1-RM strength test.

A further post-immobilisation visit will not be required for these individuals as we know from the literature that young, healthy males demonstrate a complete recovery of any loss of muscle mass and strength following a period of muscle disuse after as short a time as two weeks [30]. In a previous study (MEC 09-3-011), when volunteers return 6 weeks after a 2 week immobilisation period (natural recovery – no training administered) there is a complete restoration of muscle mass and strength. To ensure that full recovery has taken place, subject will be contacted by telephone once more at 6 weeks after cast removal.

4. STUDY POPULATION

In total, we aim to include 30 young (18-35 y) healthy males (18.5 < BMI <30 kg/m^2). Only men will be included to have a more homogenous study population, as muscle protein synthesis rates have been shown to differ between men and women [31]. The nature and the risks of the experimental procedures will be explained to all subjects and their informed consent will be obtained after approval by the local Medical Ethics Committee. All subjects will be recruited through flyers around the university campus and dedicated bulletin boards within the University of Maastricht. Subjects with a BMI above 30 or below 18.5 kg/m^2 will be excluded. These limits are based on international standards for a healthy population. Subjects wit a BMI below 18.5 will likely have a lower amount of lean body mass and may develop increased functional problems due to muscle loss caused by immobilisation. A BMI above 30 is used to exclude obese subjects. Obesity can have systemic effects, including chronic low grade inflammation. Inflammation can directly influence muscle mass and may also affect gene expression.

4.1 Inclusion criteria

- Male
- Aged from 18-35 years
- 18.5 < BMI < 30 kg/m^2

4.2 Exclusion criteria

- (Family) history of thrombosis
- Smoking
- Recent surgery (within 6 months)
- Performing regular resistance training more than once per week in the past year
- Any back/leg/knee/shoulder complaints which may interfere with the use of crutches
- Current systemic use of corticosteroids, growth hormone, testosterone, immunosuppressants or insulin
- All co-morbidities interacting with mobility and muscle metabolism of the lower limbs (e.g. arthritis, spasticity/rigidity, all neurological disorders and paralysis)
- Use of anti-coagulants
- Pre-existing renal disease or those with a potential risk for renal dysfunction (diabetes, hypertension, reduced glomerular filtration rate)

4.3 Sample size calculation

With two independent groups, the sample size (N) was calculated with a power of 80% and a significance level of 0.05 with the following formula:

$$N_1 = N_2 = (z_{0.8} + z_{0.975})^2.(\sigma_1^2 + \sigma_2^2)/(\mu_1 - \mu_2)^2$$

$z_{0.8} = 0.84$

$z_{0.975} = 1.96$

For the power calculation we used the change in quadriceps CSA determined by CT scan as our primary endpoint. Based on previous data we expect the standard deviation of the measurement of quadriceps CSA to be approximately 1.8% in both groups. Furthermore, based on previous studies performed in our group, we expect that the muscle loss from baseline in the control group will be approximately 5%. We consider a reduction in muscle loss by 40% (i.e. a total muscle CSA loss of 3% instead of 5%) as clinically relevant. The latter will likely lead to significant retention of muscle function and strength. Therefore, the power calculation is as follows:

$$N_1 = N_2 = (0.84 + 1.96)^2.(1.8^2 + 1.8^2)/(5-3)^2 = 12.7$$

Taking into consideration a drop-out rate of 10% during testing, the final number of subjects that should be recruited after screening is 15 per group. The total amount of participants we will include is 30. An exclusion rate of 25% is expected during screening (due to the use of medication, being resistance trained etc. please see exclusion criteria) therefore, we expect to screen a total of 40 subjects.

5. TREATMENT OF SUBJECTS

5.1 Investigational treatments

5.1.1 Seven days of immobilisation via a full leg cast

One leg will be immobilized at a 30 degree knee joint angle of flexion for 7 days by means of a full leg cast (see **Figure 2**). The leg to be immobilized will be randomized and balanced in the study between left and right. Prophylactic medication to prevent deep vein thrombosis (DVT) is not warranted when a single joint (in this study the knee joint) is immobilized [32]. However, to further minimize the risk of DVT development all subjects will perform daily exercises to activate the calf muscle pump. The time needed to perform these exercises is 5 minutes. Exercises will be performed 3 times a day (i.e. morning, afternoon and evening) for the 7 days of the immobilisation period. Subjects will be provided with crutches because they are not allowed to bear weight on their immobilized leg. Full instructions will be given on the correct use of crutches. Although subjects will be provided with and instructed on the use of crutches, subjects are most likely to experience a large degree of physical impairment during the 7 days of immobilisation. Activities like driving a car and/or bicycle, and sport activities will be prohibited. Other activities might require assistance (e.g. travelling, climbing stairs). Walking is only permitted with crutches, without weight bearing on the immobilized leg. Overall, it is important to note that for the duration of the immobilisation period physical mobility will be severely hampered for all subjects.

Following the removal of the cast, subjects are likely to experience some of the effects of a reduction in muscle mass and strength of their immobilized leg. Normal mobility and activities of daily living will no longer be impaired, but sports performance may be somewhat below normal level for 2-14 days. We will determine whether creatine influences the speed of recovery by measuring muscle CSA one week after the cast is removed. We expect muscle function to restore itself entirely within a few weeks after cast removal [9, 30]. Throughout the immobilisation and recovery period the researcher will establish regular contact with the participating subjects to discuss any problems or queries. During the recovery period all subjects are also at liberty to seek contact with the researcher, physiotherapist and/or physician to discuss any possible problems related to the immobilisation period.

5.1.2 Creatine supplementation

During the study, subjects will take either a creatine supplement or a placebo. More details on the supplements can be found in section 6.

5.2 Use of co-intervention

All subjects will be asked to consume a standardized meal the day before the test days (i.e. pre and post immobilisation when the CT scan is performed and the muscle biopsies are obtained). This standardized meal is an ‘Aviko maaltijdpannetje’ and will be provided to the volunteers during the screening to take home in preparation for the test day (Section 3.2 & Appendix D4.1). In addition, subjects will be asked to record their food intake and physical activity for 3 days before the start of the test days in specific diaries that will be provided during screening (Appendices F2.1 and F2.2, respectively). All volunteers will be instructed to refrain from any sort of heavy physical exercise over the course of the study and not deviate from their normal eating habits for 48 hours before the test day as well as during the 7 days of immobilisation. Subjects will also be asked to refrain from consuming alcohol in the 48 hours leading up to a test day.

.

6. INVESTIGATIONAL PRODUCT

6.1 Name and description of investigational product

Creatine monohydrate (CreaPure) will be provided by AlzChem AG. The supplement is listed as a dietary supplement; for safety documentation see appendix D4.2 and D4.3. Creatine will be provided in sachets containing 5 grams of creatine, 7.5 grams of dextrose monohydrate (appendix D4.4) and 7.5 grams of maltodextrin (appendix D4.5). Glucose is added for taste and the presence of the poorly soluble creatine will be masked by the similarly poorly soluble maltodextrin. Placebo sachets are selected to contain exactly the same ingredients, but without the creatine. Creatine contains no metabolisable energy and as such both the placebo and the creatine supplement will be isocaloric. The resulting mixtures have similar characteristics (taste and solubility), as described previously [33, 34]. Using this combination of ingredients has been used successfully in the past by our group [34]. The mixture will be odourless and slightly sweet when dissolved in water.

6.2 Summary of Findings from Non-Clinical Studies

Research on creatine over the past two decades has focused primarily on the ergogenic effects of creatine supplementation. A wide range of studies have shown that creatine is capable of enhancing the training effect of resistance exercise, improving body composition and increasing muscular strength and endurance (for reviews see [15-17]). Creatine has also been investigated for certain pathological conditions, including muscle pathologies, neurological diseases and obesity related diseases (for reviews see [35, 36]). An extensive review has been written by Stout et al. on the safety and the various effects of creatine supplementation [37].

Currently two studies have investigated the effect of creatine supplementation during immobilisation, as discussed in paragraph 1. Hespel *et al.* [13] found a positive effect of creatine supplementation on the recovery after immobilisation-induced muscle loss of the upper leg. Johnston *et al.* [14] found a significant reduction in muscle loss during one week immobilisation of the elbow. Effects on muscle mass were accompanied by positive effects on muscle strength.

6.3 Summary of known and potential risks and benefits

Creatine is a widely used supplement and has a good safety record. Clinical studies using similar dosages for similar durations or longer (i.e. up to 12 weeks) have not shown any major side effects [38, 39]. Minor side effects associated with the usage of creatine are mild nausea and gastro-intestinal discomfort. There are some concerns that creatine may have negative effects on people suffering from renal disorders, and as such these people will be

excluded from this study. Any complaints occurring during the study will be recorded and monitored thoroughly. More details on the safety of creatine can be found in chapter 12 (Structured Risk Analysis).

6.4 Description and justification of route of administration and dosage

Subjects are asked to dissolve the contents of each sachet in a glass of lukewarm water and drink the solution immediately afterwards. Creatine is provided as a powder in sachets with glucose and maltodextrine (as described in 6.1). Using capsules would require subjects to ingest 27 capsules daily during the loading phase. Providing creatine in powdered form is standard practice [27, 34, 40-47]. Dosages used in this study are based on previous studies on creatine monohydrate supplementation. For creatine loading the most commonly used dosage is 20g per day [27, 34, 40-47]. 20g of creatine per day for 5 days, taken as 4 separate portions of 5g, can increase creatine stores in muscle to a maximal level [27, 34]. Maintenance dosage, the amount of creatine needed to maintain these higher levels of creatine in the muscle, range from 2g, 5g, 10g, and up to 16g per day in previous studies [27, 34, 40-47]. We will use a maintenance dosage of 5g of creatine per day. This is a safe and effective dose and eliminates the need to use two different types of sachets for the treatment group. A dose of 3-5 g per day ('one tea spoon') is also recommended by manufacturers and is used by most athletes in daily practice.

In the week after immobilisation (recovery week), subjects will maintain their supplementation in order to determine the effects of creatine supplementation on the early recovery after immobilisation.

6.5 Dosages, dosage modifications and method of administration

Subjects will be randomly assigned to either the creatine supplemented group (CR; n=15) or the control group (PLA; n=15). Subjects assigned to the CR group will receive 5 grams of creatine monohydrate four times per day for a total of 20 grams per day in the 5 days leading up to the second test day (loading phase). Supplements will be taken spread over the day; subjects are asked to ingest one sachet with breakfast, lunch, dinner, and at ~22:00 hrs during the loading phase. This is to ensure that muscle creatine content is at maximal levels before immobilisation [26, 27, 38]. During immobilisation the daily dose is reduced to a single dose of 5 grams of creatine monohydrate per day to maintain high levels of muscular creatine content [13, 27, 38]. Daily intake of a single sachet is maintained during the recovery phase (see also figure 1). During immobilisation and recovery, sachets will be ingested with dinner only. Subjects in the PLA group will also ingest 4 sachets per day during the loading phase and 1 sachet per day during immobilisation and recovery, in exactly the same manner as the CR group. Compliance will be checked by asking the subjects to mark each separate

intake on a log form (Appendix F2.3) and by returning both empty and unused sachets to the researchers.

6.6 Preparation and labelling

Zip-lock sachets will be prepared in the kitchen of Human Biology at Maastricht University. This kitchen represents a hygienic environment specifically assigned for the preparation of food and dietary supplements. All procedures will be performed by an independent technician not involved in the study execution. All sachets will first be filled with 7.5 g of dextrose monohydrate and 7.5 g of maltodextrin, with each volume weighed separately using plastic weighing boats on a weighing scale with an accuracy of 0.01 g. An accuracy of 0.05 g (i.e. a total weight range of 7.45 - 7.55 g) will be taken into account. The latter represents an inaccuracy of less than 1%. Subsequently, for half of the sachets (i.e. only the sachets for the CR group), 5g of creatine will be added. Each sachet will be labelled with a randomization number. The independent technician will have the randomization scheme in order to ensure correct labelling of the sachets (i.e. according to the randomization over the PLA and CR group). An example of the label is shown below:

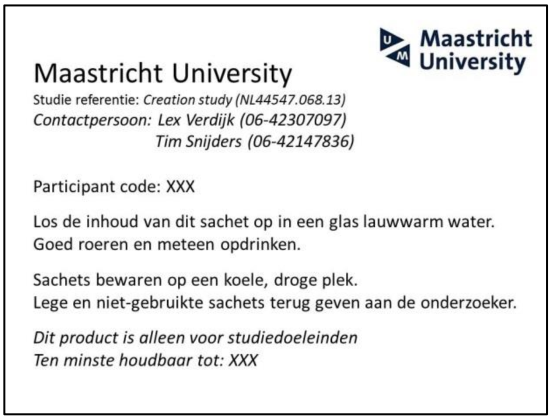
Maastricht University

Maastricht University
Studie referentie: *Creation study (NL44547.068.13)*
Contactpersoon: Lex Verdijk (06-42307097)
Tim Snijders (06-42147836)

Participant code: XXX

Los de inhoud van dit sachet op in een glas lauwwarm water.
Goed roeren en meteen opdrinken.

Sachets bewaren op een koele, droge plek.
Lege en niet-gebruikte sachets terug geven aan de onderzoeker.

Dit product is alleen voor studiedoeleinden
Ten minste houdbaar tot: XXX

For each subject, 40 sachets will be prepared (note that 36 sachets are needed for the study). After manufacturing, the contents of both PLA and CR sachets will be checked by determining the concentration of the supplement when in solution. This will be done by randomly taking 3 PLA and 3 CR sachets and dissolving the contents. The analysis will be performed at the department of Human Movement Sciences. The analytical report of the content determination of the sachets (both creatine and carbohydrate content) will be handed in to the ethical committee before commencement of the study. Should the procedure be divided over 2 production rounds, then the analysis on content will be repeated for the second batch.

All sachets will be labelled and stored in a sealed box per participant (similar labels as shown above will be on the box), in a cool and dry place until distribution to the study participants takes place. The involved researchers are in charge of storage and distribution of the supplements after manufacturing by the independent technician. Distribution logs will be kept and subjects are asked to return both empty and unused sachets in order to check compliance with the protocol. Supplement sachets for the first week will be provided on the first test day, supplement sachets for the second and third week will be provided on the casting day and the day of cast removal, respectively. The expiration date for the ingredient with the shortest shelf life will be regarded as the expiration date for the supplements and will

be printed on the label. Note that the study is expected to last approximately 6 months (i.e. excluding the analysis) and the shortest shelf life is beyond 1 year (up to 3 y for creatine).

7. METHODS

7.1 Study parameters/endpoints

7.1.1 Main study parameter/endpoint

The main study endpoint is quadriceps muscle CSA. This will be measured by CT scan.

7.1.2 Secondary study parameters/endpoints

Secondary endpoints include: whole upper leg muscle CSA (measured by CT scan); muscle fiber type specific CSA and muscle fiber type-specific satellite cell content (all determined by immuno-histochemistry and fluorescence microscopy); Gene expression is measured using whole genome Affymatrix microarrays. Special focus is on the changes in genes involved in anabolic signaling, muscle catabolism and satellite cell proliferation and differentiation. Metabolomics (both muscle and blood) will be applied to determine potential new biomarkers for muscle loss. Muscle strength will be determined by 1RM testing.

7.1.3 Plasma and serum samples

Within this study, four blood samples (18 ml each; 10 ml for plasma, 8 ml for serum) will be obtained by venipuncture. The first samples are taken before creatine supplementation starts, two samples are taken pre- and post-immobilisation, and the last sample is taken after 1 week of recovery. A total of 72 ml of venous blood will be taken over the study. Aliquots of plasma and serum will be frozen in liquid nitrogen and stored at –80°C until analysis.

7.1.4 Muscle biopsies

A single muscle biopsy will be obtained from the designated leg to be immobilized before supplementation starts, during the pre and post immobilisation test days and a week after the cast has been removed. Per subject we will take a total of four muscle tissue samples. Biopsies will be obtained from the middle region of the *m. vastus lateralis* muscle (15 cm above the patella) and approximately 2 cm below entry through the fascia by the percutaneous needle biopsy technique described by Bergström et al. [28], using a modified needle (Maastricht Instruments). With this instrument, better results are obtained for sample size, and subjects' discomfort is minimized. In short, skin and muscle fascia will be locally anesthetized using 1% xylocaine. After 10 minutes a small incision will be made in the skin and fascia after which the biopsy needle will be introduced in the muscle. Vacuum will be applied to the needle and, with the needle kept *in situ*; <u>a small muscle sample (total ± 70-150 mg) will be taken</u>. Following the muscle biopsy, the skin will be closed using a Steristrip® and covered by Tegaderm® after which a pressure bandage will be applied (Acrylastic®).

The percutaneous needle biopsy technique was introduced within the Department of Human Movement Sciences by Profs. Hans Keizer and Harm Kuipers more than 25 years ago. Since this introduction, the technique has been successfully used to perform numerous human *in vivo* studies focusing on skeletal muscle metabolism within our Department (for review see [48, 49]). In addition, recently approved MEC proposals continue to use the muscle biopsy technique in young (MEC 09-3-080, MEC 09-3-054) and elderly men (MEC 07-3-086, MEC 09-3-080, MEC 09-3-054, MEC 09-3-078, MEC 10-3-065, MEC 10-3-80). Based on our extensive previous experience, discomfort for the subjects with the technique used in our laboratory is negligible.

7.1.5 Other study parameters

Other study parameters include plasma glucose and insulin concentrations, age, body weight, body height, BMI and leg volume.

7.2 Randomization, blinding and treatment allocation

In this study, all eligible subjects will be allocated (1:1 ratio) to the CR or the PLA group in a randomized manner. Based on the order in which subjects enter the study, a unique participant code is assigned. These participant codes are linked to either the CR group or PLA group using a computer random number generator. All randomization and blinding procedures will be executed by an independent scientist. Supplement sachets will be coded with the unique participant codes. Subjects will be randomly assigned to one of the groups by a researcher not involved in the study via a random-number generator. A random number generator will also be used by an independent scientist when designating a leg for immobilisation.

7.3 Study procedures

7.3.1 Screening & pre-testing

- Medical questionnaire will be filled in.
- Body weight and height will be measured, and leg volume will be determined.
- 1RM will be estimated
- Use of crutches will be explained and practiced.

7.3.2 Diet and activity prior to testing

- Subjects will consume a standardized meal (*Aviko maaltijdpannetje*) the evening before the test day.
- All volunteers will be instructed to refrain from any sort of heavy physical exercise 2 d before the test day

- All volunteers will be instructed to keep their diet as constant as possible for 48h before the test day. A constant diet should be maintained prior to test days to avoid any unanticipated effects of diet on the measured variables in the biopsy sample (e.g. mRNA/protein expression of key signalling proteins). Subjects will be asked to record their food intake and physical activity for 3 days before the start of the test day.

7.3.3 Experimental test days (3x)

- Subjects will report to the laboratory at 8.00 am in an overnight fasted state
- A CT-scan will be performed as described previously [50].
- A single blood draw and muscle sample will be obtained.
- 1RM will be tested

Please see section 3 and Figure 1, for a total overview of the study protocol.

7.3.4 Analyses

Quadriceps muscle CSA will be determined from CT-scans, as described previously [51].

Circulating levels of glucose and insulin will be measured in plasma. Insulin and glucose are common basic measurements and provide information on for example whether a subject is in a fasted state or not. Cortisol, vitamin D, testosterone, and IGF-1 will be measured in plasma or serum when appropriate. Cortisol is an important catabolic stress hormone and influences muscle mass. IGF-1 and testosterone are anabolic hormones and can also affect muscle size [52]. Vitamin D can influence gene expression in the muscle [53]. These analyses require approximately 1.5 mL of plasma and 1.5 mL of serum. Metabolome analysis requires an additional 1mL of serum and plasma. Aliquots of plasma and serum will be frozen in liquid nitrogen and stored at –80°C until analysis, with the remainder (if applicable) being stored for any additional measurements.

Muscle samples will be cut into 5 µm thick cross sections and will be stained for immuno-histochemical determination of fiber type distribution, fiber type specific CSA, myonuclear content and satellite cell content [50, 54]. The latter will provide necessary information on the cellular mechanisms underlying the potential reduction in muscle mass. Histological analysis will require approximately 20-30mg of muscle tissue. Creatine and phosphocreatine content will be determined biochemically as described in detail previously [33, 34] and will require ±20mg of muscle tissue. Changes in gene expression mediated by creatine supplementation and immobilisation are measured using whole genome Affymatrix microarrays. Special focus is on the changes in genes involved in anabolic signalling, muscle catabolism and satellite

cell proliferation and differentiation. Both metabolomics and transcriptomics analyses require 10-20mg of muscle tissue, each. Any remaining muscle tissue will be stored for possible future analyses or re-analysis.

Total energy intake and macronutrient composition of the diet will be determined from the dietary intake records. In addition, physical activity level will be assessed by analyzing the physical activity diaries.

7.4 Withdrawal of individual subjects

Subjects can leave the study at any time for any reason if they wish to do so without any consequences. The investigator can decide to withdraw a subject from the study for urgent medical reasons.

7.5 Replacement of individual subjects after withdrawal

In case of withdrawal, the subject will not be replaced. A 10% drop out rate has been included in the sample size calculation.

7.6 Follow-up of subjects withdrawn from treatment

All subjects in this study are included for follow-up after withdrawal or completion of the study. One week afterwards, subjects will be called by the coordinating investigator to monitor general recovery from the procedures. A final follow-up call will be made 6 weeks after cast removal to check whether full recovery has taken place.

7.7 Premature termination of the study

Premature termination of the study does not need any form of explanation by the subject. Severe physical discomfort, noticed by the principal investigator, coordinating investigator or subjects themselves will lead to immediate termination of the study.

8. SAFETY REPORTING

Section 10 WMO event

In accordance to section 10, subsection 1, of the WMO, the investigator will inform the subjects and the reviewing accredited METC if anything occurs, on the basis of which it appears that the disadvantages of participation may be significantly greater than was foreseen in the research proposal. The study will be suspended pending further review by the accredited METC, except insofar as suspension would jeopardise the subjects' health. The investigator will take care that all subjects are kept informed.

8.1 Adverse and serious adverse events

Adverse events are defined as any undesirable experience occurring to a subject during the study, whether or not considered related to [the investigational product / the experimental treatment]. All adverse events reported spontaneously by the subject or observed by the investigator or his staff will be recorded.

A serious adverse event is any untoward medical occurrence or effect that at any dose:

- results in death;
- is life threatening (at the time of the event);
- requires hospitalisation or prolongation of existing inpatients' hospitalisation;
- results in persistent or significant disability or incapacity;
- is a congenital anomaly or birth defect;
- is a new event of the trial likely to affect the safety of the subjects, such as an unexpected outcome of an adverse reaction, lack of efficacy of an IMP used for the treatment of a life threatening disease, major safety finding from a newly completed animal study, etc.

All SAEs will be reported through the web portal *ToetsingOnline* to the accredited METC that approved the protocol, within 15 days after the sponsor has first knowledge of the serious adverse reactions..

SAEs that result in death or are life threatening should be reported expedited. The expedited reporting will occur not later than 7 days after the responsible investigator has first knowledge of the adverse reaction. This is for a preliminary report with another 8 days for completion of the report.

8.2 Follow-up of adverse events

All adverse events will be followed until they have abated, or until a stable situation has been reached. Depending on the event, follow up may require additional tests or medical procedures as indicated, and/or referral to the general physician or a medical specialist. SAE's need to be reported till the end of study in the Netherlands, as defined in the protocol.

9. STATISTICAL ANALYSIS

All data will be presented as means ± SEM. The baseline values of the two groups (i.e. weight, height, BMI, body composition, strength, quadriceps CSA) will be compared independently against one another (i.e. CR vs. PLA) using an independent samples t-test to ensure that they were correctly randomized and that no baseline differences exist. Differences in quadriceps CSA, muscle fiber size, satellite cell content and strength between pre- and post-immobilisation will be analyzed using a repeated measures analysis of variance (ANOVA) with time (pre vs. post) as within subjects factor and treatment (CR vs. PLA) as between subjects factor. For the recovery period, post-immobilisation vs. post 1 wk recovery will be used as within subjects factor. Similarly, repeated measures ANOVA with multiple time points (i.e. pre-supplementation, pre-immobilisation, post-immobilisation, and post 1 wk recovery) will be used to determine changes over time in muscle and/or blood creatine levels. In case of missing values we will use linear mixed models; missing values will not be replaced. Should there be any significant differences between groups at baseline we will use ANCOVA with the baseline characteristics as covariates. Statistical significance will be set at $P<0.05$. Data will be analyzed with IBM SPSS version 19.0.

Analysis of the whole genome expression data will be performed using a Bayesian linear regression model (implanted in the software package LIMMA; www.bioconductor.org). Raw microarray data will be normalized using RMA quantile normalization. Statistically significant changed genes will be identified by Student paired t-test, followed by a false discovery rate correction (FDR). The latter is used to correct for multiple testing.

10. ETHICAL CONSIDERATIONS

10.1 Regulation statement

This study will be conducted according to the principles of the declaration of Helsinki (version 9, October 2008) and in accordance with the Medical Research Involving Human Subjects Act (WMO).

10.2 Recruitment and consent

All subjects will be recruited through flyers situated around the campus and on dedicated bulletin boards within the University of Maastricht. In order to ascertain that the participants in this study are well informed, both written and oral explanation of the procedures of this study will be provided by the principal investigator. After all information is provided, subjects will be given at least one week to consider their decision before their informed consent is obtained. An independent MD (M.Poeze) will be assigned to this project, who can be contacted by the subjects in case they want further information.

10.3 Benefits and risks assessment, group relatedness

The risks involved in participating in this experiment are minimal.

The incision made for obtaining the muscle biopsy will be performed by an experienced physician and will heal completely. Within our research group we have extensive experience with taking muscle biopsies. There is a small risk of infection and the muscle biopsy can in lead to minor hematoma. During the blood draw there is a small risk of fainting or haematoma. These risks are minimized by using trained and experienced personnel for taking the muscle biopsies and blood draws. Adequate pressure will be applied after the blood draw and biopsies to minimise the risk of hematoma.

The Aviko vacuum-packed and pre-weighed meals are normal food products and have been cleared for human consumption. There are no complications associated with the procedure of a single slice lower limb CT scan. The level of radiation emitted is very low; approximately 0.053 mSv per scan. In this study, subjects will undergo three CT scans and will therefore be subjected to 0.159 mSV of radiation. These scans are done routinely in studies by our group (e.g. see MEC 06-3-062, 11-3-073, 12-3-012). For comparison, the present study will provide a 25 times lower dose of radiation than the ordinary background radiation in the Netherlands (i.e. 2.5 mSv/year) and a similar level to that which one would be exposed to during a two week winter sports holiday.

In terms of time investment, the screening visit will take 2-3 hours of the subject’s time, as will the pre-supplementation, the pre-immobilisation and post-immobilisation visits. An

additional burden will be the requirement for subjects to fill in a 3 day food and activity diary (see appendix F2.1 and F2.2) before the pre and post immobilisation visits. However, the greatest inconvenience in terms of time and also mobility will be the 7 days of one-legged immobilisation.

One leg will be immobilized for seven days by means of a full leg cast at a 30 degree angle of flexion (**see Figure 2**). In a healthy population deep vein thromboses (DVT) will develop in 1 out every 1000 individuals a year [55]. After surgery and/or bone fracture immobilisation of two or more joints will increase the risk of developing DVT. The incidence of DVT occurrence during limb immobilisation following surgery and/or bone fracture is estimated between ~0.2 and ~17% [32, 56-59]. However, in the present study prophylactic medication to prevent DVT is not warranted when a single joint is immobilized [32, 56], particularly for a time period of only 7 days. However, to further minimize the risk of DVT development, subjects with any family history of thrombosis will be excluded (please see exclusion criteria; section 4.3) and all subjects will perform daily exercises to activate the calf muscle pump. The time needed to perform these exercises is 5 minutes. Exercises will be performed 3 times a day (i.e. morning, afternoon and evening) for the 7 day immobilisation period. Finally, throughout the immobilisation period all subjects will be monitored closely by the research team, who will establish regular contact with the subjects, to identify early signs of DVT development and act accordingly. Thus, in this study the overall risk of DVT is minimal (<0.1%).

The leg cast will cause a large degree of physical impairment. Subjects will be provided with crutches because they are not allowed to bear any weight on their immobilized leg. Full instructions will be given by a physiotherapist on the correct use of crutches. To minimize the risk of any injury due to this loss of physical mobility, activities like driving a car and/or bicycle, and sport activities will be prohibited for the 7 day immobilisation period. Other activities might require assistance (e.g. travelling, climbing stairs). Walking is only permitted with crutches, without weight bearing on the immobilized leg. During the first days/week after removal of the leg cast subjects will probably encounter the effects of the reduced muscle mass and strength of their immobilized leg. Normal mobility and activities of daily living will no longer be impaired, but sports performance will be somewhat below normal level in the first 2-14 days. However, since only healthy subjects will participate in this study, muscle function will most likely completely restore itself within a few weeks after cast removal [30, 49]. Indeed, it has been shown that even after 3 weeks of leg immobilisation in young individuals, the resumption of spontaneous activity for 2 weeks results in the recovery of 90% of muscle strength and 95% of muscle fiber size [30]. Subjects will be called one week after the termination of the study (i.e. 2 weeks after the immobilisation period) to monitor the

rehabilitation and evaluate the recovery. Any complaints will be discussed with the responsible physician and when necessary, specific advice will be given or further referral to the general practitioner will take place. A final follow-up phone call will be performed 6 weeks after cast removal to confirm that full recovery has taken place. If this is not the case, further follow up will be performed or referral to the general practitioner will take place when appropriate.

As discussed in section 6 and 12, the use of the creatine supplement is widely accepted for scientific and clinical purpose. No major risks are associated with the use of creatine, even for longer periods or when using higher dosages than in the current study, in healthy volunteers. Although possible, gastrointestinal problems and cramps are rare and may be based on anecdotal evidence or improper creatine usage. Water retention (leading to weight gain) is possible in the first week(s) of supplementation. More detailed information is included in paragraph 12.

10.4 Compensation for injury

The sponsor/investigator has a liability insurance which is in accordance with article 7, subsection 6 of the WMO.

The sponsor (also) has an insurance which is in accordance with the legal requirements in the Netherlands (Article 7 WMO and the Measure regarding Compulsory Insurance for Clinical Research in Humans of 23th June 2003). This insurance provides cover for damage to research subjects through injury or death caused by the study.

1. € 450.000,-- (i.e. four hundred and fifty thousand Euro) for death or injury for each subject who participates in the Research;
2. € 3.500.000,-- (i.e. three million five hundred thousand Euro) for death or injury for all subjects who participate in the Research;
3. € 5.000.000,-- (i.e. five million Euro) for the total damage incurred by the organisation for all damage disclosed by scientific research for the Sponsor as ‘verrichter’ in the meaning of said Act in each year of insurance coverage.

The insurance applies to the damage that becomes apparent during the study or within 4 years after the end of the study.

10.5 Incentives (if applicable)

Subjects will receive €225 for completion of the study. Travel expenses will be covered separately. If the subject does not complete the entire study, he will receive payment according to ratio of the study completed. If the subject drops out after the screening but

before the start of the test day, he will not receive any compensation (except for travel expenses).

11. ADMINISTRATIVE ASPECTS AND PUBLICATION

11.1 Handling and storage of data and documents

To protect the privacy of the participants, all collected data will be encoded numerically (i.e. not directly traceable to the subjects) and only the principal investigators will have access to the encoding key and the acquired data. All subjects will be offered full access to their own data and the outcomes of the project will be explained to them. The CT scans will only be analyzed in the context of the present study (i.e. to determine muscle CSA) and as such, potential abnormalities may be missed.

All subject data will be kept for a maximum of 15 years, and (with approval of the subject), all human material will be stored also be stored for a maximum of 15 years for potential future analysis in the line of this research, after which all material will be destroyed.

11.2 Amendments

All amendments to the protocol (i.e. regarding the intervention itself as well as the different measurements and/or tests performed) will be notified to the METC. Substantial amendments will be submitted for approval before they will be implemented in the study protocol. Annual reports will be submitted to the METC regarding progress of the study, number of subjects included/completed, and serious adverse events. Upon ending of the study, the METC will be notified within 8 weeks.

11.3 Annual progress report

The sponsor/investigator will submit a summary of the progress of the trial to the accredited METC once a year. Information will be provided on the date of inclusion of the first subject, numbers of subjects included and numbers of subjects that have completed the trial, serious adverse events/ serious adverse reactions, other problems, and amendments.

11.4 End of study report

The investigator will notify the accredited METC of the end of the study within a period of 8 weeks. The end of the study is defined as the last subject's last visit.

In case the study is ended prematurely, the investigator will notify the accredited METC, including the reasons for the premature termination.

Within one year after the end of the study, the investigator/sponsor will submit a final study report with the results of the study, including any publications/abstracts of the study, to the accredited METC.

11.1 Public disclosure and publication policy

The results of this investigation will be published in a high-impact, scientific journal, regardless of the outcome of this study. All subjects will be given insight into the (individual) results of this study.

12. STRUCTURED RISK ANALYSIS

12.1 Potential issues of concern

12.1.1 Muscle cramps

Muscle cramps are commonly attributed to creatine supplementation. Because creatine supplementation alters the fluid content in muscle tissue some have suggested that creatine disrupts electrolyte balance. Various studies that have investigated creatine supplementation have failed to report any increased incidence of muscle cramping in relation to creatine usage [60-62], even when a daily dosage of 15.75g of creatine is used for 28 days [17]. A review of the safety of creatine has stated that cramps are likely not caused by the ingestion of additional creatine [63]. The link between muscle cramps and creatine usage is based largely on anecdotal evidence [64].

12.1.2 Gastrointestinal complaints

Reports on gastrointestinal complaints caused by creatine supplementation are inconsistent. One study reported a relatively high incidence of problems where 3 out of 9 subjects showed gastrointestinal problems [60]. In this study subjects were supplemented with 40g creatine per day, combined with 400mg caffeine. Another study reported diarrhoea in 31% of the subjects using 6-8g creatine per day [65]. However, other studies showed no gastrointestinal problems associated with the use of creatine [17, 66, 67]. With the exception of the abovementioned studies, gastrointestinal complaints associated with creatine supplementation are mostly anecdotal [64, 68]. There may be an explanation for these problems: others have suggested that gastrointestinal problems arise when creatine monohydrate is not dissolved completely or when more than 10g of creatine is taken in a single serving due to an unusually high osmotic load [69].

12.1.3 Kidney problems

A case study published in the Lancet reported an possible link between creatine usage and renal dysfunction [70]. A later study has shown that this incident could be explained by an interaction between cyclosporine and creatine [68, 71]. Several studies have investigated whether creatine has a negative impact on kidney health and none of these have reported any significant negative effects [67, 72, 73]. A negative effect on kidney health has been largely refuted according to several reviews on the safety of creatine [63, 74, 75]. Because exogenous creatine ingestion is still an added load on the kidneys it is generally recommended to avoid creatine supplementation in people who have impaired kidney function [74].

12.1.4 Water retention

Creatine is known to cause weight gain of 1-3kg in the first week(s) of supplementation. This is mostly water. Weight will go back to normal after creatine supplementation is stopped, except for any changes in muscle mass [68].

12.1.5 Dosage

In our study we have a 5 day loading phase where 5g of creatine is taken 4 times per day. Afterwards we have a maintenance phase of 16 days where 5g creatine is taken per day. Many studies have used the same dosage (20g / day) for the loading phase [27, 34, 40-47], and daily maintenance dosages of 2g [27, 34], 5g [41, 44], 7g [43], 10g [40, 42, 45-47] and 15.75g [17] have been used previously. None of these studies have reported any significant side-effects, other than water retention mentioned above.

12.2 Synthesis

There have been no reported serious issues with creatine supplementation. Problems commonly associated with creatine supplementation, cramps and gastrointestinal distress, are largely based on anecdotal evidence and there is no consistent evidence in previous studies that these side-effects are common. We use 5g of creatine in a single serving to avoid any problems potentially associated with large single doses of creatine. Study participants are instructed to dissolve the sachets completely. We expect that this will limit any negative gastrointestinal difficulties. We exclude anyone with a pre-existing kidney disease or anyone who may have impaired kidney function. The dosage used in this study has been used previously in several studies without any negative side effects, with the exception of the two studies mentioned above [60, 65]. Some studies have used even higher daily dosages without problems. Furthermore, the relative short-term nature of the present study (with only 3 weeks in total of supplementation) is unlikely to cause any serious complaints. Of course, any complaints occurring during the study will be monitored thoroughly.

13. REFERENCES

1. Deitrick, J., *The effect of immobilization on metabolic and physiological functions of normal men.* Bull N Y Acad Med, 1948. **24**(6): p. 364-75.
2. Gibson, J., et al., *Decrease in human quadriceps muscle protein turnover consequent upon leg immobilization.* Clin Sci (Lond), 1987. **72**(4): p. 503-9.
3. Stuart, C., et al., *Bed-rest-induced insulin resistance occurs primarily in muscle.* Metabolism, 1988. **37**(8): p. 802-6.
4. Blotner, H., *Effect of prolonged physical inactivity on tolerance of sugar.* Arch Intern Med, 1945. **75**: p. 39-44.
5. Tzankoff, S. and A. Norris, *Effect of muscle mass decrease on age-related BMR changes.* J Appl Physiol, 1977. **43**(6): p. 1001-6.
6. Blanc, S., et al., *Energy and water metabolism, body composition, and hormonal changes induced by 42 days of enforced inactivity and simulated weightlessness.* J Clin Endocrinol Metab., 1998. **83**(12): p. 4289-97.
7. Appell, H.J., *Muscular atrophy following immobilisation. A review.* Sports Medicine, 1990. **10**(1): p. 42-58.
8. Adams, G.R., V.J. Caiozzo, and K.M. Baldwin, *Skeletal muscle unweighting: spaceflight and ground-based models.* J Appl Physiol, 2003. **95**(6): p. 2185-201.
9. Berg, H.E., et al., *Effects of lower limb unloading on skeletal muscle mass and function in humans.* J Appl Physiol, 1991. **70**(4): p. 1882-5.
10. Blakemore, S.J., et al., *Effects of limb immobilization on cytochrome c oxidase activity and GLUT4 and GLUT5 protein expression in human skeletal muscle.* Clin Sci (Lond), 1996. **91**(5): p. 591-9.
11. Christensen, B., et al., *Short-term immobilization and recovery affect skeletal muscle but not collagen tissue turnover in humans.* J Appl Physiol, 2008. **105**(6): p. 1845-51.
12. White, M.J., C.T. Davies, and P. Brooksby, *The effects of short-term voluntary immobilization on the contractile properties of the human triceps surae.* Q J Exp Physiol, 1984. **69**(4): p. 685-91.
13. Hespel, P., et al., *Oral creatine supplementation facilitates the rehabilitation of disuse atrophy and alters the expression of muscle myogenic factors in humans.* J Physiol, 2001. **536**(Pt 2): p. 625-33.
14. Johnston, A.P.W., et al., *Effect of Creatine Supplementation During Cast-Induced Immobilization on the Preservation of Muscle Mass, Strength, and Endurance.* The Journal of Strength & Conditioning Research, 2009. **23**(1): p. 116-120 10.1519/JSC.0b013e31818efbcc.
15. Paddon-Jones, D., E. Børsheim, and R.R. Wolfe, *Potential Ergogenic Effects of Arginine and Creatine Supplementation.* The Journal of Nutrition, 2004. **134**(10): p. 2888S-2894S.
16. Francaux, M. and J.R. Poortmans, *Effects of training and creatine supplement on muscle strength and body mass.* European Journal of Applied Physiology and Occupational Physiology, 1999. **80**(2): p. 165-168.
17. Kreider, R.B., et al., *Effects of creatine supplementation on body composition, strength, and sprint performance.* Medicine and Science in Sports Exercise, 1998. **30**(1): p. 73-82.
18. Tarnopolsky, M.A., et al., *Creatine monohydrate enhances strength and body composition in Duchenne muscular dystrophy.* Neurology, 2004. **62**(10): p. 1771-1777.
19. Willoughby, D.S. and J.M. Rosene, *Effects of oral creatine and resistance training on myogenic regulatory factor expression.* Medicine and science in sports and exercise, 2003. **35**(6): p. 923-929.
20. Parise, G., et al., *Effects of acute creatine monohydrate supplementation on leucine kinetics and mixed-muscle protein synthesis.* Journal of Applied Physiology, 2001. **91**(3): p. 1041-1047.
21. Wall, B.T. and L.J. van Loon, *Nutritional strategies to attenuate muscle disuse atrophy.* Nutr Rev, 2013. **71**(4): p. 195-208.
22. Olsen, S., et al., *Creatine supplementation augments the increase in satellite cell and myonuclei number in human skeletal muscle induced by strength training.* The Journal of Physiology, 2006. **573**(2): p. 525-534.

23. Burke, D.G., et al., *Effect of creatine supplementation and resistance-exercise training on muscle insulin-like growth factor in young adults.* International journal of sport nutrition and exercise metabolism, 2008. **18**(4): p. 389-398.
24. Deldicque, L., et al., *Increased IGF mRNA in human skeletal muscle after creatine supplementation.* Medicine and science in sports and exercise, 2005. **37**(5): p. 731-736.
25. Psilander, N., R. Damsgaard, and H. Pilegaard, *Resistance exercise alters MRF and IGF-I mRNA content in human skeletal muscle.* Journal of Applied Physiology, 2003. **95**(3): p. 1038-1044.
26. Terjung, R.L., et al., *American College of Sports Medicine roundtable. The physiological and health effects of oral creatine supplementation.* Med Sci Sports Exerc, 2000. **32**(3): p. 706-17.
27. Hultman, E., et al., *Muscle creatine loading in men.* J Appl Physiol., 1996. **81**(1): p. 232-7.
28. Bergstrom, J., *Percutaneous needle biopsy of skeletal muscle in physiological and clinical research.* Scand J Clin Lab Invest, 1975. **35**(7): p. 609-16.
29. Verdijk, L.B., et al., *One-repetition maximum strength test represents a valid means to assess leg strength in vivo in humans.* J Sports Sci, 2009. **27**(1): p. 59-68.
30. Hortobagyi, T., et al., *Changes in muscle strength, muscle fibre size and myofibrillar gene expression after immobilization and retraining in humans.* J Physiol, 2000. **524 Pt 1**: p. 293-304.
31. Smith, G.I., et al., *Differences in muscle protein synthesis and anabolic signaling in the postabsorptive state and in response to food in 65-80 year old men and women.* PLoS One, 2008. **3**(3): p. e1875.
32. Metz, R., E.M.M. Verleisdonk, and G.J.M.G. van der Heijden, *Insufficient evidence for routine use of thromboprophylaxis in ambulatory patients with an isolated lower leg injury requiring immobilization: results of a mete-analysis.* Eur J Trauma Emerg Surg, 2009. **35**: p. 169-175.
33. van Loon, L.J., et al., *Creatine supplementation increases glycogen storage but not GLUT-4 expression in human skeletal muscle.* Clin Sci (Lond), 2004. **106**(1): p. 99-106.
34. van Loon, L.J., et al., *Effects of creatine loading and prolonged creatine supplementation on body composition, fuel selection, sprint and endurance performance in humans.* Clin Sci (Lond), 2003. **104**(2): p. 153-62.
35. Pearlman, J.P. and R.A. Fielding, *Creatine Monohydrate as a Therapeutic Aid in Muscular Dystrophy.* Nutrition Reviews, 2006. **64**(2): p. 80-88.
36. Gualano, B., et al., *Exploring the therapeutic role of creatine supplementation.* 2010. **38**(1): p. 31-44.
37. Stout, J., et al., *Creatine Consumption in Health*, in *Essentials of Creatine in Sports and Health*2008, Humana Press. p. 127-172.
38. Poortmans, J.R. and M. Francaux, *Adverse effects of creatine supplementation: fact or fiction?* Sports Med, 2000. **30**(3): p. 155-70.
39. Robinson, T.M., et al., *Dietary creatine supplementation does not affect some haematological indices, or indices of muscle damage and hepatic and renal function.* Br J Sports Med, 2000. **34**(4): p. 284-8.
40. Arciero, P.J., et al., *Comparison of creatine ingestion and resistance training on energy expenditure and limb blood flow.* Metabolism, 2001. **50**(12): p. 1429-1434.
41. Bemben, M.G., et al., *Creatine supplementation during resistance training in college football athletes.* Medicine and science in sports and exercise, 2001. **33**(10): p. 1667-1673.
42. Jówko, E., et al., *Creatine and β-hydroxy-β-methylbutyrate (HMB) additively increase lean body mass and muscle strength during a weight-training program.* Nutrition, 2001. **17**(7): p. 558-566.
43. Bermon, S., et al., *Effects of creatine monohydrate ingestion in sedentary and weight-trained older adults.* Acta physiologica scandinavica, 1998. **164**: p. 147-156.
44. Kelly, V.G. and D.G. Jenkins, *Effect of oral creatine supplementation on near-maximal strength and repeated sets of high-intensity bench press exercise.* The Journal of Strength & Conditioning Research, 1998. **12**(2): p. 109-115.

45. Peeters, B.M., C.D. Lantz, and J.L. Mayhew, *Effect of oral creatine monohydrate and creatine phosphate supplementation on maximal strength indices, body composition, and blood pressure.* The Journal of Strength & Conditioning Research, 1999. **13**(1): p. 3-9.
46. Stout, J., et al., *Effects of 8 weeks of creatine supplementation on exercise performance and fat-free weight in football players during training.* Nutrition Research, 1999. **19**(2): p. 217-225.
47. Stout, J.R., et al., *Effects of Twenty-Eight Days of Beta-Alanine and Creatine Monohydrate Supplementation on the Physical Working Capacity at Neuromuscular Fatigue Threshold.* Journal of strength and conditioning research: the research journal of the NSCA, 2006. **20**(4): p. 928-931.
48. Koopman, R., et al., *Nutritional interventions to promote post-exercise muscle protein synthesis.* Sports Med, 2007. **37**(10): p. 895-906.
49. Koopman, R. and L.J.C. van Loon, *Aging, exercise and muscle protein metabolism.* J Appl Physiol, 2009.
50. Verdijk, L., et al., *Skeletal muscle hypertrophy following resistance training is accompanied by a fiber type-specific increase in satellite cell content in elderly men.* J Gerontol A Biol Sci Med Sci, 2009. **64**(3): p. 332-9.
51. Leenders, M., et al., *Protein Supplementation During Resistance-Type Exercise Training in the Elderly.* Med Sci Sports Exerc, 2012.
52. Glass, D.J., *Signaling pathways perturbing muscle mass.* Curr Opin Clin Nutr Metab Care, 2010. **13**(3): p. 225-9.
53. Hamilton, B., *Vitamin D and human skeletal muscle.* Scand J Med Sci Sports, 2010. **20**(2): p. 182-90.
54. Verdijk, L.B., et al., *Skeletal muscle hypertrophy following resistance training is accompanied by a fiber type-specific increase in satellite cell content in elderly men* J Gerontol A Biol Sci Med Sci, 2009.
55. Ageno, W., et al., *Epidemiology and risk factors of venous thromboembolism.* Semin Thromb Hemost, 2006. **32**(7): p. 651-8.
56. Glover, E., et al., *Immobilization induces anabolic resistance in human myofibrillar protein synthesis with low and high dose amino acid infusion.* J Physiol, 2008. **586**: p. 6049-61.
57. Ettema, H.B., et al., *Prevention of venous thromboembolism in patients with immobilization of the lower extremities: a meta-analysis of randomized controlled trials.* J Thromb Haemost, 2008. **6**(7): p. 1093-8.
58. Nokes, T.J. and J. Keenan, *Thromboprophylaxis in patients with lower limb immobilisation - review of current status.* Br J Haematol, 2009.
59. Testroote, M., et al., *Low molecular weight heparin for prevention of venous thromboembolism in patients with lower-leg immobilization.* Cochrane Database Syst Rev, 2008(4): p. CD006681.
60. Vandenberghe, K., et al., *Long-term creatine intake is beneficial to muscle performance during resistance training.* Journal of Applied Physiology, 1997. **83**(6): p. 2055-2063.
61. Greenwood, M., et al., *Creatine supplementation during college football training does not increase the incidence of cramping or injury.* Molecular and cellular biochemistry, 2003. **244**(1-2): p. 83-88.
62. Gotshalk, L.A., et al., *Creatine supplementation improves muscular performance in older men.* Medicine and science in sports and exercise, 2002. **34**(3): p. 537-543.
63. Dalbo, V.J., et al., *Putting to rest the myth of creatine supplementation leading to muscle cramps and dehydration.* British Journal of Sports Medicine, 2008. **42**(7): p. 567-573.
64. Poortmans, J.R. and M. Francaux, *Adverse Effects of Creatine Supplementation: Fact or Fiction?* Sports Medicine, 2000. **30**(3): p. 155-170.
65. Juhn, M.S., J.W. O'Kane, and D.M. Vinci, *Oral creatine supplementation in male collegiate athletes: a survey of dosing habits and side effects.* Journal of the American Dietetic Association, 1999. **99**(5): p. 593.

66. Green, A.L., et al., *Carbohydrate ingestion augments skeletal muscle creatine accumulation during creatine supplementation in humans.* American Journal of Physiology - Endocrinology And Metabolism, 1996. **271**(5): p. E821-E826.
67. Poortmans, J.R. and M. Francaux, *Renal dysfunction accompanying oral creatine supplements.* The Lancet, 1998. **352**(9123): p. 234.
68. Persky, A.M. and G.A. Brazeau, *Clinical Pharmacology of the Dietary Supplement Creatine Monohydrate.* Pharmacological Reviews, 2001. **53**(2): p. 161-176.
69. Ostojic, S.M. and Z. Ahmetovic, *Gastrointestinal distress after creatine supplementation in athletes: are side effects dose dependent?* Research in Sports Medicine, 2008. **16**(1): p. 15-22.
70. Pritchard, N. and P. Kalra, *Renal dysfunction accompanying oral creatine supplements.* The Lancet, 1998. **351**(9111): p. 1252-1253.
71. Tran, T.T., W. Dai, and H.K. Sarkar, *Cyclosporin A Inhibits Creatine Uptake by Altering Surface Expression of the Creatine Transporter.* Journal of Biological Chemistry, 2000. **275**(46): p. 35708-35714.
72. Poortmans, J.R. and M. Francaux, *Long-term oral creatine supplementation does not impair renal function in healthy athletes.* Medicine and science in sports and exercise, 1999. **31**: p. 1108-1110.
73. Kreider, R., et al., *Long-term creatine supplementation does not significantly affect clinical markers of health in athletes.* 2003. **244**(1-2): p. 95-104.
74. Kim, H., et al., *Studies on the safety of creatine supplementation.* 2011. **40**(5): p. 1409-1418.
75. Salomons, G., et al., *Safety of Creatine Supplementation*, in *Creatine and Creatine Kinase in Health and Disease*2008, Springer Netherlands. p. 275-289.
