# Supplementary material for: Nandrolone decanoate administration does not attenuate muscle atrophy during a short period of disuse
Source: PLoS One. 2019 Jan 28;14(1):e0210823. doi: 10.1371/journal.pone.0210823 (PMC6349315; doi:10.1371/journal.pone.0210823)
Supplement: S2 File — (PDF) [file pone.0210823.s003.pdf]

The effect of Nandrolone Decanoate Injection and Leucine Supplementation on Muscle Loss During Immobilisation

The Effect of Leucine and Nandrolone Supplementation on Muscle Loss During Immobilisation

**Protocol ID**	**NL50679.068.15**
**Short title**	**Leucine and Nandrolone and disuse atrophy**
**Version**	**2**
**Date**	**28-04-2015**
**Coordinating investigator/project leader**	Astrid Horstman, PhD<br>Human Movement Sciences<br>MUMC+<br>astrid.horstman@maastrichtuniversity.nl<br>+31-43-3881394<br><br>Evelien Backx, MSc<br>Human Movement Sciences<br>MUMC+<br>Evelien.backx@maastrichtuniversity.nl<br>+31-43-3881810
**Principal investigators**	Prof. Luc van Loon, PhD<br>Human Movement Sciences<br>MUMC+<br>l.vanloon@maastrichtuniversity.nl<br>+31-43-3881397
**Sponsor**	NUTRIM School for Nutrition, Toxicology and Metabolism, UM
**Responsible physician**	Irene Fleur Kramer, MD<br>Human Movement Sciences<br>MUMC+<br>Irenefleur.Kramer@maastrichtuniversity.nl<br>+31-43-3882215
**Independent physician(s)**	Fred Hartgens, MD, PhD<br>Capgroep Epidemiologie en Algemene Chirurgie, Polikliniek Sportgeneeskunde<br>MUMC+<br>f.hartgens@maastrichtuniversity.nl<br>+31-43-3881428
**Laboratory sites**	UM (Human Movement Sciences)

PROTOCOL SIGNATURE SHEET

Name	Signature	Date
**Coordinating Investigator / Project Leader:**<br><br>Dr. Astrid Horstman<br>(Post-doc, Human Movement Sciences, MUMC+)<br><br>Evelien Backx, MSc<br>(PhD student, Human Movement Sciences, MUMC+)		
**Principal investigators:**<br><br>Prof. L.J.C. van Loon<br>(Professor, Human Movement Sciences, MUMC+)		

TABLE OF CONTENTS

1. INTRODUCTION AND RATIONALE 9
2. OBJECTIVES 12
3. STUDY DESIGN 13
3.1 Screening (first visit) 13
3.2 Diet and activity prior to testing 14
3.3 Pre and post immobilisation test days 15
3.4 Immobilisation protocol (third visit) 16
4. STUDY POPULATION 18
4.1 Inclusion criteria 18
4.2 Exclusion criteria 18
4.3 Sample size calculation 19
5. TREATMENT OF SUBJECTS 20
5.1 Investigational treatments 20
5.1.1 Seven days of immobilisation via a full leg cast 20
5.1.1 Nandrolone decanoate and leucine supplementation 20
5.2 Use of co-intervention 20
6. INVESTIGATIONAL PRODUCT 22
6.1 Name and description of investigational product 22
6.2 Summary of Findings from Non-Clinical Studies 22
6.3 Summary of known and potential risks and benefits 23
6.4 Description and justification of route of administration and dosage 23
6.5 Dosages, dosage modifications and method of administration 24
6.6 Preparation and labelling 24
7. METHODS 25
7.1 Study parameters/endpoints 25
7.1.1 Main study parameter/endpoint 25
7.1.2 Secondary study parameters/endpoints 25
7.1.3 Plasma and serum samples 25
7.1.4 Muscle biopsies 25
7.1.5 Other study parameters 26
7.2 Randomization, blinding and treatment allocation 26
7.3 Study procedures 26
7.3.1 Screening & pre-testing 26
7.3.2 Diet and activity 27
7.3.3 Experimental test days (2x) 27
7.3.4 Analyses 27
7.4 Withdrawal of individual subjects 28
7.5 Replacement of individual subjects after withdrawal 28
7.6 Follow-up of subjects withdrawn from treatment 28
7.7 Premature termination of the study 28
8. SAFETY REPORTING 29

Section 10 WMO event 29
8.1 Adverse and serious adverse events....29
8.2 Follow-up of adverse events....30
9. STATISTICAL ANALYSIS 31
10. ETHICAL CONSIDERATIONS....32
10.1 Regulation statement 32
10.2 Recruitment and consent....32
10.3 Benefits and risks assessment, group relatedness 32
10.4 Compensation for injury 34
10.5 Incentives (if applicable)....34
11. ADMINISTRATIVE ASPECTS AND PUBLICATION 35
11.1 Handling and storage of data and documents 35
11.2 Amendments....35
11.3 Annual progress report....35
11.4 End of study report....35
11.1 Public disclosure and publication policy....36
12. STRUCTURED RISK ANALYSIS....37
12.1 Potential issues of concern....37
12.2 Synthesis 37
13. REFERENCES 38

LIST OF ABBREVIATIONS AND RELEVANT DEFINITIONS

**AE**	Adverse Event
**AR**	Adverse Reaction
**BMI**	Body Mass Index
**CSA**	Cross-sectional area
**CT**	Computed Tomography
**DVT**	Deep Vein Thrombosis
**EudraCT**	European drug regulatory affairs Clinical Trials
**IC**	Informed Consent
**IGF-1**	Insulin-like Growth Factor 1
**IMP**	Investigational Medicinal Product
**METC**	Medical research ethics committee (MREC); in Dutch: medisch ethische toetsing commissie (METC)
**SAE**	Serious Adverse Event
**Sponsor**	The sponsor is the party that commissions the organisation or performance of the research, for example a pharmaceutical company, academic hospital, scientific organisation or investigator. A party that provides funding for a study but does not commission it is not regarded as the sponsor, but referred to as a subsidising party.
**WMO**	Medical Research Involving Human Subjects Act (in Dutch: Wet Medisch-wetenschappelijk Onderzoek met Mensen
**1-RM**	One-repetition maximum

SUMMARY

Rationale: Muscle loss can occur in several situations, for example with aging or during inactivity because of illness or injury. This muscle loss is associated with several negative consequences, such as impaired physical functioning, reduced insulin sensitivity, and an increase in fat mass. For these reasons, attenuating muscle loss during short-term disuse is of importance. Possible strategies to alleviate muscle loss are to supplement with nandrolone decanoate (ND) or leucine (Leu). We will investigate nandrolone decanoate's and leucine's ability to attenuate muscle loss, both from a physiological and mechanistic standpoint, during short-term knee-immobilization.

Objective: To determine the attenuating effect of ND injection and Leu supplementation on muscle mass loss during short-term immobilisation in healthy, young men.

Study design: randomized, placebo-controlled, parallel-arm study.

Study population: 30 young healthy volunteers (age 18-35 years old, 18.5 < BMI < 30 kg/m^2).

Intervention: Seven days of one-legged knee immobilisation in young subjects with ND injection or Leu supplementation. The ND group receives 200 mg at the first day of immobilisation. Leucine will be supplemented for 1 week (7,5 g/d), starting at the first day of immobilisation. A randomly selected leg is immobilized for seven days. Muscle mass and strength will be determined and muscle biopsies will be collected before and immediately after immobilisation.

Main study parameters/endpoints:

Primary: quadriceps muscle cross-sectional area (CSA), measured by single-slice CT scan.

Secondary: Leg muscle strength (1-RM), type I and II muscle fiber size and satellite cell content, whole thigh muscle CSA and expression and phosphorylation status of proteins and myogenic regulatory factors.

Nature and extent of the burden and risks associated with participation, benefit and group relatedness:

The risks involved in participating in this experiment are minimal. Muscle biopsies will be taken through a small (5-10 mm) incision, following local anaesthetics of the skin and muscle fascia. The muscle tissue will heal completely. Muscle biopsies will only be obtained by an experienced physician, minimizing the risk for infection and local hematoma. Seven days of limb immobilisation via a full leg cast will impair subject's mobility for this period and may increase the risk of deep vein thrombosis. To minimize any risk of injury, subjects will not be allowed to drive a vehicle or ride a bicycle and will have regular contact with the investigators. The seven day immobilisation period will lead to a loss of muscle mass and strength. However, the expected loss of muscle mass and strength following immobilisation

will be rapidly (<4 weeks) regained due to the inclusion of only healthy volunteers. The CT scans used to measure changes in muscle CSA will expose study participants to a low level of radiation (0.159 mSV in total). No side effects have been reported for leucine. Side effects of nandrolone injections have been reported using higher doses and much longer interventions: increased libido, hair loss, acne, rash, itch, nausea, muscle soreness, malaise, oedema, increased blood pressure, decreased liver function, bruise or swelling at the injection place, hoarseness, increased prostate or penis, longer and/or sometimes painful erections and disturbance in forming of sperm cells.

1. INTRODUCTION AND RATIONALE

Muscle loss can occur for several reasons, such as inactivity because of illness or (sports) injuries, illnesses themselves or simply old age. A decrease in muscle mass can have a profound impact on sports performance, functional performance, and quality of life, as it can lead to decreased strength and power, but also insulin resistance, lower basal metabolic rate and obesity [1-4]. One way to induce muscle loss and study its effects is immobilisation. Previous studies have shown that immobilisation of the knee can rapidly induce muscle atrophy [5-11]. Previous studies performed at MUMC+ have shown that immobilisation can cause a reduction of 3.5% in quadriceps muscle cross-sectional area in as little as five days in young men (MEC 11-3-073, [12]). Correspondingly, muscle strength is substantially reduced in these subjects (i.e. ~10%). As such, immobilisation is a suitable model to induce loss of muscle mass and strength and, therefore, can be used to test nutritional strategies to attenuate muscle loss during situations of muscle disuse and/or injury. Several compounds have shown promise regarding the protection of muscle mass in catabolic situations. Amongst them are leucine and nandrolone decanoate.

Leucine

Leucine is a branched-chain amino acid (BCAA). Leucine is recognized to have a particular role in the regulation of muscle protein synthesis [13-16] and can reduce the rate of muscle protein degradation [17]. Ingestion of additional leucine as a supplement with a meal may stimulate specific intracellular pathways associated with muscle protein synthesis. There is evidence for a leucine-mediated increase in plasma insulin [18], resulting in a regulation of the ribosomal protein S6 kinase-1 (S6K1) and the eukaryotic initiation factor (eIF)-4E-binding protein-1 (4E-BP1), which are involved in the initiation of muscle protein synthesis [19{Kimball, 2006 #34]}. Moreover, there is evidence suggesting that plasma leucine concentrations regulate muscle protein synthesis by insulin-independent mechanisms [20]. Studies have shown that the stimulatory effect of leucine supplementation on protein synthesis also occurs at the level of protein translation initiation and involves signalling through the mammalian Target Of Rapamycin (mTOR) [21]. This protein kinase seems to serve as a convergence point for leucine- and insulin-mediated effects on translation initiation. As such, leucine can stimulate skeletal muscle protein synthesis through both insulin-dependent as well as insulin-independent mechanisms.

Consequently, leucine supplementation has been tested to limit muscle atrophy during immobilization based on the hypothesis that increasing leucine intake could overcome anabolic resistance. However, leucine supplementation was mainly tested in immobilized

animals. Several animal studies are done on leucine supplementation as a nutritional strategy for muscle recovery *after* unloading [22-24]. Leucine supplementation *before* and/or *during* immobilization, however, seems to be really promising. Young rats received leucine for 3 days before immobilization and during 7 days of immobilization. With the supplemented diet, skeletal muscle wasting was attenuated via inhibition of ubiquitin ligases [25]. Moreover, hindlimb suspension in rats has been shown to increase MAFbx/Atrogin-1and MuRF1 proteins, which play a pivotal role in various muscle atrophies. Branched-chain amino acid attenuated the increase in atrogin-1 and MuRF1 in rat soleus muscles [26]. In humans, dietary free leucine has been demonstrated to be particularly efficient at stimulating muscle protein synthesis during ageing [27-31], but dietary free leucine supplementation cannot reverse the lack of recovery of muscle mass *after* prolonged immobilization during ageing [22]. The preventative effect of leucine on muscle loss in humans *during* immobilisation remains to be established.

Nandrolone decanoate

Nandrolone, with nandrolone decanoate as one of its esters [32-35], is an anabolic steroid. After intramuscular injection it binds to the androgen receptor and it has, like testosterone, both an anabolic and an androgenic activity. IGF-1 expression and the Akt/mTORC signaling in muscles is stimulated (i.e. increased muscle protein synthesis) and expression of FoxO transcription (a proxy for muscle proteolysis) is attenuated. Nandrolone decanoate had been shown to be able to modulate proliferation and adhesion of myoblasts in vitro [36] and to stimulate proliferation of satellite cells. Moreover, nandrolone treatment was shown to increase satellite cell numbers in chicken pectoralis muscle [37].

In human studies nandrolone decanoate has been shown to improve lean body mass, for instance in body builders [38], HIV-infected men [39{Batterham, 2001 #63, 40-43], COPD [44], patients with unresectable non-small cell lung cancer [45] and menopausal, osteoporotic women [46]. In rats, nandrolone decanoate has been shown to have a muscle-enhancing effect during unloading. Treatment with the steroid prevented atrophy and functional changes induced by 3 weeks of unweighting in rat skeletal muscles (preceeded by 5 [47] or 6 [48] weeks of nandrolone decanoate treatment under normal conditions). However, whether nandrolone decanoate will attenuate muscle loss during immobilisation in humans as well, has not been investigated yet.

The present study is a proof-of-principle study to determine the potential of ND and leucine to attenuate muscle loss during immobilisation, as well as the underlying mechanisms. The findings can be directly translated to healthy young individuals (e.g. athletes) that suffer from injury for which immobilisation is needed. Indirectly, insight from this study may reveal

potential targets for further study in clinical conditions, such as supplementing leucine or injecting ND in more compromised elderly and/or hospitalized patients. In order to study the underlying mechanisms of disuse muscle atrophy, and the potential beneficial impact of ND and leucine hereon, we intend to immobilize the knee of 30 young subjects for seven days. Subjects will receive ND (n=15) or leucine (n=15) to determine its efficacy to attenuate the loss of muscle mass and strength during disuse. Additionally, we want to investigate whether these compounds influence the early recovery following immobilisation. In addition to determining the physiological/functional effects of immobilisation and ND injection and leucine supplementation on muscle mass and function), we intend to establish the compounds' mechanisms of action on (potentially) attenuating muscle mass.

2. OBJECTIVES

The primary aim of this study is to determine the effect of nandrolone decanoate injection and leucine supplementation on muscle mass loss during short-term immobilisation in healthy, young individuals. In addition, we aim to study the underlying mechanisms of nandrolone decanoate injection and leucine supplementation during disuse muscular atrophy.

We intend to test the following hypotheses:

1. Nandrolone decanoate injection reduces muscle loss in the upper leg during immobilisation
2. Leucine supplementation during immobilisation of the knee reduces muscle loss in the upper leg

In addition, we intend to answer the following research questions:

1. Which genes and pathways are up- or down-regulated in skeletal muscle tissue by leucine supplementation or nandrolone decanoate injection during immobilisation?
2. Which genes are up- or down-regulated in skeletal muscle tissue by immobilisation?
3. What is the effect of leucine supplementation and nandrolone decanoate injection during immobilisation on muscle fiber size and satellite cell content?

3. STUDY DESIGN

The present study will use a randomised, placebo controlled, parallel-arm study design with two groups. All volunteers (n=30) will be subjected to 7 days of one-legged knee immobilisation by means of a full leg cast, either with nandrolone decanoate injection (n=15, nandrolone decanoate group) or leucine supplementation (n=15, leucine group).

The nandrolone decanoate group will be given 4 ml with 200 mg nandrolone at the first day of the unloading period. The leucine supplementation will consist of 7.5 g leucine per day; i.e. 3*2.5 g to be supplemented with each main meal (breakfast, lunch, and dinner) during immobilisation.

Previous studies performed within our research group have shown that five days of immobilisation can induce a loss in muscle CSA of approximately 3.5% in young individuals (i.e. ~ 0.7% per day, MEC 11-3-073). Previous studies have shown that muscle loss during immobilisation is rapid and linear during the first week. This has been demonstrated by our group (MEC 11-3-073 and 09-3-011) and by studies performed at other universities as was recently reviewed [49]. Consequently, we expect the decrease in CSA to be approximately 5% after the seven days of immobilisation in our study. We have slightly increased the duration of immobilisation from 5 to 7 days to limit the number of subjects needed to show a benefit of nandrolone decanoate and leucine supplementation.

Primary measurement is CT scan, secondary measurements are 1-RM leg strength test and blood and muscle samples. All measurements are performed at baseline (before immobilization) and immediately after immobilization. Figure 1 presents an overview of the study outline.

3.1 Screening (first visit)

Prior to the study, volunteers' suitability to participate will be assessed during a single screening session. After explaining all procedures, written informed consent will be obtained from subjects willing to participate. We will determine body weight, height and blood pressure. Subjects will be asked to fill in a medical questionnaire enquiring about their general health, medical history, use of medication and sports activities. During the screening we will go over the medical questionnaire with the subjects and let them sign it. This questionnaire will be carefully assessed by the responsible physician before subjects are allowed entry into the study.

Maximal muscle strength will be estimated on the screening day (visit 1) using a one-repetition maximum estimation as done before. This estimate will be used for a 1-RM

confirmation where the actual strength is determined. During the 1-RM test, subjects will be asked to take place on a Leg Extension machine. By raising the weight that is lifted after every set, eventually the weight that can be lifted only once is determined; this is the subject's One Repetition Maximum (1-RM). The 1-RM test will be performed with both legs separately to determine single-legged 1-RM.

Volunteers will be instructed on the proper use of crutches which will be necessary during the immobilisation period. In the event of any unexpected medical findings during the screening, subjects will always be notified. If a subject does not want to receive this notification he cannot participate in the study. Our policy on co-incidental findings is that we will inform the subjects' general practitioner about the results that were found. If subjects do not want this, they cannot participate in the study. Following screening and acceptance for the study, volunteers will be given an unique randomization number, which automatically allocates each individual to one of the two experimental groups.

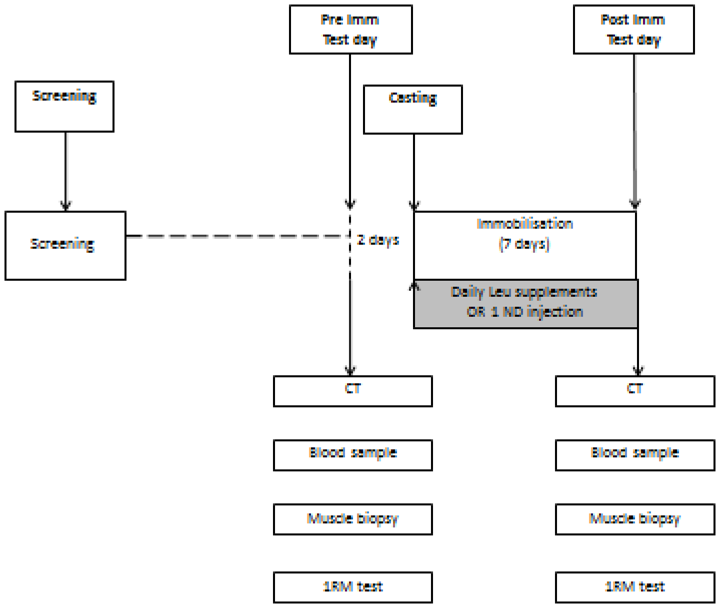

Figure 1: Overview of the study design. Imm: immobilisation, Leu: leucine, ND: nandrolone decanoate.

3.2 Diet and activity prior to testing

All subjects will consume a standardized dinner the evening before the test day. This standardized dinner is an 'Aviko maaltijdpannetje' and will be purchased at a regular supermarket in Maastricht. The expiration date from the manufacturer will be checked. The

meals will be stored in an appropriate freezer of the ‘dietary-kitchen’ at the department of Human Biology. The subjects will receive the meal before each test. The precise composition and preparation methods are described in Appendix D4.1. The subjects will be instructed to store the meal in a freezer until preparation and to prepare the meal themselves according to the instructions on the label. Subjects will be instructed to refrain from any sort of heavy physical exercise over the course of the study and not deviate from their normal eating habits. In addition, subjects will be asked to record their food intake and physical activity for 3 days before the first data collection test day in specific diaries. Subjects will also be asked to refrain from consuming alcohol in the 48 hours before a test day.

3.3 Pre and post immobilisation test days

One test day will be performed prior to immobilisation and two test days will be done afterwards. These sessions last approximately 3 hours. The first test day will be carried out two days prior to casting. Subjects will arrive at the laboratory at 08.00 am in the fasted state having not consumed anything (except water) since the controlled meal (described above, section 3.2) latest at 10.00 pm the previous evening. During this session a 1-RM-strength test and a CT scan will be performed, and a blood sample and muscle biopsy will be taken. We will also measure blood pressure.

First, a single slice CT scan (IDT 8000; Philips Medical Systems, Best, Netherlands) at 15 cm above the base of the patella will be performed in the Academic Hospital Maastricht (Department of Radiology) to determine (anatomical) cross sectional area (CSA) of the *quadriceps femoris* muscle of both legs. While the participants will be supine with their legs extended and their feet secured, a 3-mm thick axial image will be taken. The scanning characteristics will be as follows: 120kV, 300mA, rotation time of 0.75 sec, and a field of view of 500 mm. The exact scanning position will be measured and marked with a semi-permanent marker for replication.

Thereafter, a muscle biopsy will be obtained from the leg to be immobilized. The muscle biopsy will be obtained from the middle region of the *m. vastus lateralis* (15 cm above the patella and approximately 2 cm away from the fascia) by the percutaneous needle biopsy technique [50]. Muscle biopsies will be carefully freed from any visible non-muscle tissue. Part of the sample will be rapidly frozen in liquid nitrogen-cooled isopentane, embedded in Tissue-Tek (Sakura Finetek Europe BV, The Netherlands). The remaining part will be frozen immediately in liquid nitrogen. Muscle biopsies will be stored at –80 ºC for subsequent analysis. The leg chosen to obtain the muscle biopsy from and, consequently, the one to be immobilized, will be randomized. A single blood draw will also be obtained by venipuncture. Subjects will receive a breakfast after samples have been taken.

Subsequently, 1-RM strength will be determined. After warming up, the load will be set lower than the estimated 1-RM (obtained during screening), and increased after each successful lift until failure as described previously [51]. Rest periods of 1-2 minutes will be allowed between attempts. A repetition is valid if the subject uses proper form and is able to complete the entire lift in a controlled manner without assistance.

3.4 Immobilisation protocol (third visit)

Two days following the pre immobilisation test day, a 7 day period of immobilisation will commence. Volunteers will arrive at the laboratory at 8am. The muscle biopsy wound will be inspected carefully by the responsible physician and with her approval the leg will be immobilized with a cast: from 10 cm above the ankle to half way the upper leg (30 degree flexion of the knee-joint; see Fig 2). Next, the subject will be instructed again on how to use crutches and to not bear weight on the casted limb, and to minimize muscle contractions of the upper leg. This protocol has been already successfully used for previous immobilisation studies (METC 09-3-011, METC 11-3-073, METC 12-3-012, METC 12-3-063) and for the Creation study that is currently running in our lab (METC 13-3-023). We will use the data from the placebo group from that study (METC 13-3-023). All those participants have signed an informed consent and have given agreed permission to store their body material for 15 years after finishing this study. These samples can be used for further analyses as described in the information for subjects.

A previous immobilisation study (MEC 09-3-011) has employed the same full leg cast protocol for a period of two weeks as a means of immobilizing the leg. Thus, the present study will pose much less of a burden given the shorter duration of immobilisation (7 days).

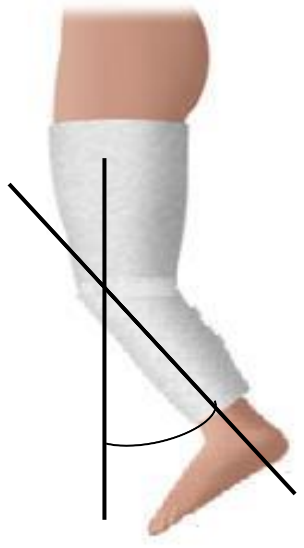

Figure 2: Representation of a full leg cast at a knee angle of 30 degrees of flexion

During the seven days of immobilisation, all subjects will be checked upon regularly by a member of the research team. These check-ups will serve to answer any questions/concerns from the participant and to check compliance with the protocol. If the subject has no questions or problems in the first two days, the research team will contact him every other day. All subjects are at liberty to seek contact with the researcher and/or physician to discuss any possible problems related to the immobilization or participation in the study.

The morning after the seven days of immobilisation, volunteers will come to the university for the post immobilisation visit. First, the cast will be removed and then the volunteer will not be allowed to place any weight on the leg. Instead, the subject will be transported in a wheelchair to the CT scanner where a single slice CT scan will be performed as described above. Thereafter, subjects will be transported by wheelchair to the laboratory where another muscle biopsy and blood sample will be collected as described above. Thus, the biopsy will be collected while the subject is still in the immobilized state (i.e. before they have performed any weight bearing muscle contractions). After breakfast, when the subject sits down, blood pressure will be measured. Following the muscle biopsy and blood sample, 1RM strength testing will be performed as described above.

A further post-immobilisation visit will not be required for these individuals as we know from the literature that young, healthy males demonstrate a complete recovery of any loss of muscle mass and strength following a period of muscle disuse after as short a time as two weeks [52, 53]. In previous immobilization studies (MEC 09-3-011; MEC 11-3-073), volunteers returned 6 weeks after a 2 week immobilisation period (natural recovery – no training administered) and showed a complete restoration of muscle mass and strength [12]. To ensure that full recovery has taken place, subjects will be contacted by telephone once more at 6 weeks after cast removal.

4. STUDY POPULATION

In total, we aim to include 30 young (18-35 y) healthy males (18.5 < BMI <30 kg/m^2). Only men will be included to have a more homogenous study population, as muscle protein synthesis rates have been shown to differ between men and women [54]. The nature and the risks of the experimental procedures will be explained to all subjects and their informed consent will be obtained during the screening. Recruitment starts after the study is approved by the local Medical Ethics Committee. All subjects will be recruited through flyers around the university campus and dedicated bulletin boards within the University of Maastricht. Moreover, dependent on responses, advertisements will be placed at local newspapers, at websites of local study- or student associations and digi-prik.nl.

4.1 Inclusion criteria

- Male
- Aged from 18-35 years
- 18.5 < BMI < 30 kg/m^2

4.2 Exclusion criteria

- (Family) history of thromboembolic events
- Smoking
- Recent surgery (within 6 months prior to the study)
- Performing progressive resistance training more than three times per week in the past year
- Any back/leg/knee/shoulder complaints which may interfere with the use of crutches
- Current systemic use of corticosteroids, anabolic steroids, growth hormone, testosterone, nandrolone, protein supplements, immunosuppressants or insulin, blood sugar decreasing medication or EPO
- All co-morbidities interacting with mobility and muscle metabolism of the lower limbs (e.g. arthritis, spasticity/rigidity, all neurological disorders and paralysis)
- Use of anti-coagulants
- Pre-existing renal disease or those with a potential risk for renal dysfunction (diabetes, hypertension, reduced glomerular filtration rate)
- Liver disease
- Heart failure
- Migraine
- Allergy to nuts or soy
- High blood pressure (>140 mmHg systolic and >90 mmHg diastolic)

In case of doubt, in- or exclusion of subject will be discussed with responsible physician or principal investigator.

4.3 Sample size calculation

With two independent groups, the sample size (N) was calculated with a power of 80% and a significance level of 0.05 with the following formula:

$$N_1 = N_2 = (z_{0.8} + z_{0.975})^2 . (\sigma_1^2 + \sigma_2^2) / (\mu_1 - \mu_2)^2$$

$z_{0.8} = 0.84$
$z_{0.975} = 1.96$

For the power calculation we used the change in quadriceps CSA determined by CT scan as our primary endpoint. Based on previous data we expect the standard deviation of the measurement of quadriceps CSA to be approximately 1.8% in both groups. Furthermore, based on previous studies performed in our group, we expect that the muscle loss from baseline in the control group will be approximately 5%. We consider a reduction in muscle loss by 40% (i.e. a total muscle CSA loss of 3% instead of 5%) as clinically relevant. The latter will likely lead to significant retention of muscle function and strength. Therefore, the power calculation is as follows:

$$N_1 = N_2 = (0.84 + 1.96)^2 . (1.8^2 + 1.8^2) / (5 - 3)^2 = 12.7$$

Taking into consideration a drop-out rate of 10% during testing, the final number of subjects that should be recruited after screening is 15 per group. The total amount of participants we will include is 30. An exclusion rate of 50% is expected during screening (due to the use of medication, being resistance trained etc. please see exclusion criteria) therefore, we expect to screen a total of 60 subjects.

5. TREATMENT OF SUBJECTS

5.1 Investigational treatments

5.1.1 Seven days of immobilisation via a full leg cast

One leg will be immobilized at a 30 degree knee joint angle of flexion for 7 days by means of a full leg cast (see **Figure 2**). The leg to be immobilized will be randomized and balanced in the study between left and right. Prophylactic medication to prevent deep vein thrombosis (DVT) is not warranted when a single joint (in this study the knee joint) is immobilized [55]. However, to further minimize the risk of DVT development all subjects will perform daily exercises to activate the calf muscle pump. The time needed to perform these exercises is 5 minutes. Exercises will be performed 3 times a day (i.e. morning, afternoon and evening) for the 7 days of the immobilization period. Subjects will be provided with crutches because they are not allowed to bear weight on their immobilized leg. Full instructions will be given on the correct use of crutches. Although subjects will be provided with and instructed on the use of crutches, subjects are most likely to experience a large degree of physical impairment during the 7 days of immobilization. Activities like driving a car and/or bicycle, and sport activities will be prohibited. Other activities might require assistance (e.g. travelling, climbing stairs). Walking is only permitted with crutches, without weight bearing on the immobilized leg.

After the removal of the cast, subjects are likely to experience some of the effects of a reduction in muscle mass and strength of their immobilized leg. Normal mobility and activities of daily living will no longer be impaired, but subjects are not allowed to do any heavy physical exercise until the last visit. After the last visit, sports performance might be somewhat below normal level. Comparing our results to the placebo group from MEC 13-3-023, we expect muscle function to restore itself entirely within a few weeks after cast removal [52, 56].

5.1.1 Nandrolone decanoate and leucine supplementation

During the study, subjects will either get a nandrolone decanoate injection or leucine supplements. More details on the supplements can be found in section 6.

5.2 Use of co-intervention

All subjects will be asked to consume a standardized meal the day before the test days (i.e. pre and post immobilization when the CT scan is performed and the muscle biopsies are obtained). This standardized meal is an ‘Aviko maaltijdpannetje’ and will be provided to the volunteers during the screening to take home in preparation for the test day. In addition, subjects will be asked to record their food intake and physical activity for 3 days before the

start of the test days in specific diaries that will be provided during screening (Appendices F2.1 and F2.2, respectively). All volunteers will be instructed to refrain from any sort of heavy physical exercise over the course of the study and not deviate from their normal eating habits for 48 hours before the test day as well as during the 7 days of immobilization. Subjects will also be asked to refrain from consuming alcohol in the 48 hours leading up to a test day.

.

6. INVESTIGATIONAL PRODUCT

6.1 Name and description of investigational product

ASPEN (Aspen Pharma Trading Limited 3016 Lake Drive Citywest Business Campus Dublin 24, Ireland) is the Marketing Authorisation Holder. Nandrolone decanoate is listed as an anabolic steroid and will be provided in 1 ml syringes with 50mg nandrolone-17β-decanoaat (see Patient Information Leaflet (PIL) in attachment). The EudraCT information (2015-000578-37) is attached.

Free leucine (BUFA, Uitgeest, The Netherlands) will be provided in capsules (0.5 grams of leucine powder per capsule). Five capsules must be consumed during each main meal (3 times a day).

6.2 Summary of Findings from Non-Clinical Studies

An in vitro study on **leucine** has clearly demonstrated myofibrillar and not generic protein accretion in skeletal muscle following leucine supplementation, and suggests this involves pre-translational control of MyHC expression by leucine [57]. Due to the promising results from animal studies indicating that meals supplemented with leucine improve the postprandial muscle protein synthesis in old rats [29] there has been an increasing amount of studies that aim to assess the efficacy of leucine supplementation to increase muscle mass *in vivo* in healthy humans [28, 58]. These studies seem to indicate that supplementation with leucine has beneficial effects on muscle protein synthesis (for a review, see [59]. Although long-term leucine supplementation does not increase muscle mass in healthy elderly or elderly type 2 diabetic men [60, 61], it is unknown what leucine does in attenuating muscle loss during unloading in young healthy men.

Research on **nandrolone** over the past two decades has focused primarily on the ergogenic effects of nandrolone supplementation [62-64]. Several studies describe pharmacokinetics and pharmacodynamics of nandrolone esters [32, 34]. A wide range of studies have shown that nandrolone is capable of further enhancing the training effect of resistance exercise, improving body composition and increasing muscular strength [38, 41, 65, 66{Kuipers, 1993 #98, 67]} and plays a positive in muscle recovery [67-71]. Nandrolone has also been shown to improve lean body mass in certain pathological conditions, including HIV [39-43, 72], COPD [44, 73, 74], non-small cell lung cancer [45], menopausal, osteoporotic women [46, 75],knee surgery [69], dialysis [76] and unloading [47, 48].

6.3 Summary of known and potential risks and benefits

The ingested **leucine** is part of the normal diet as a precursor for protein synthesis and imposes no risk. Higher doses per day for a much longer period of time have been safely used before (e.g. [77]).

Nandrolone is a widely used supplement and has a good safety record. Clinical studies using similar dosages for similar durations or longer (i.e. up to 12 weeks) have not shown any major side effects [41{Chlebowski, 1986 #56, 64]}. Side effects of nandrolone supplementation have been reported, depending on dose and sensitivity: increase libido, hair loss, acne, rash, itch, nausea, muscle soreness, malaise, oedema, increased blood pressure, decreased liver function, bruise or swelling at the injection place, hoarseness, increased prostate or penis, longer, sometimes painful erections and disturbance in forming of sperm cells.

Bagchus et al [32] describe safety parameters of nandrolone decanoate: “A total of 36 subjects (66.7%) reported AEs in this study, and none of the subjects reported an SAE. The most frequently reported AE was headache, which was reported by 18.5% of the subjects (20%, 24%, and 12% after 50, 100, and 150 mg, respectively) followed by rhinitis (14.8%), back pain (14.8%), and rash (11.1%). Most of these events were of mild intensity. In all cases, the relation to study drug was judged unlikely or none. There were 13 drug-related AEs reported in 10 subjects: testicular pain (one subject), injection site pain (one subject), injection site reaction (one subject), back pain (one subject), fatigue (five subjects), hot flushes (one subject), and leg pain (one subject). The intensity of all drug-related AEs was assessed as mild. Local tolerance was good. No itching, swelling, or bruising was observed after injection. Two subjects showed mild redness directly after dosing. Pain at the injection site was reported in 13 subjects 2 h after injection. Mild pain at the injection site was still present in one subject in each dose group 24 h after injection.”

More details on the safety of nandrolone can be found in chapter 12 (Structured Risk Analysis).

6.4 Description and justification of route of administration and dosage

200 mg nandrolone will be injected intramuscularly in *m. gluteus maximus*. This dose has been injected for 12 weeks in patients with HIV (Mulligan 2001) and for 8 weeks in body builders (van Marken Lichtenbelt, 2004). Also considerably higher doses (600mg/week) have been safely given for 12 weeks in eugonadal HIV positive men (Sattler 1999). All studies resulted in a significant increase in lean body mass. Based on the dose response study of Bagchus (2005) we expect the peak in serum concentrations after a 200mg injection after 5 or 6 days. Wijnand et al have described the pharmacokinetic parameters of nandrolone after

intramuscular administration (200mg) of nandrolone decanoate (Deca-Durabolin) to healthy volunteers (3865478).

Leucine is provided in capsules. This way of providing leucine is standard practice and dosages used in this study are based on previous studies on leucine supplementation [60, 61]#78}.

6.5 Dosages, dosage modifications and method of administration

Subjects will be randomly assigned to either the nandrolone decanoate (ND) group or the leucine (Leu) group. Subjects assigned to the ND group will receive 200 mg nandrolone decanoate at the first day of immobilisation. Leucine supplements will be taken spread over the day; subjects are asked to ingest 5 capsules (5*0.5 = 2.5 g leucine) during breakfast, five during lunch and five during dinner. Subjects of this study are compared with the placebo group of a parallel study (METC 13-3-023) who have given permission to store their body material for 15 years after finishing this study and “These samples can be used for further analyses as described in the information for subjects.” Compliance will be checked by asking the subjects to mark each separate intake on a log form (Appendix F2.3) and by counting both empty and unused sachets that are returned to the researchers.

6.6 Preparation and labelling

Leucine capsules will be produced externally. Labeling, see appendix D3.1.
Nandrolone decanoate will be delivered in 1ml vials, containing 100 mg/ml Deca-Durabolin, solution for injection (Appendix D2).

7. METHODS

7.1 Study parameters/endpoints

7.1.1 Main study parameter/endpoint

The main study endpoint is quadriceps muscle CSA. This will be measured by single-slice CT scan.

7.1.2 Secondary study parameters/endpoints

Secondary endpoints include: whole thigh muscle CSA (measured by CT scan); muscle fibre type-specific CSA and muscle fibre type-specific satellite cell content (all determined by immuno-histochemistry and fluorescence microscopy). Muscle strength will be determined by 1-RM testing. The expression and phosphorylation status of proteins and myogenic regulatory factors (specified in 7.3.4) will be established via Western blots. Quantitative Real-Time PCR Analysis of MAFbx/Atrogin-1, MuRF1, FoxO and Ubiquitin Expression will be done. Activation of NF-κB will be measured in serum by the ELISA technique. Antibody against Pax7 and immunocytochemical techniques will be used to identify satellite cells and myonuclei.

7.1.3 Plasma and serum samples

Within this study, two blood samples (18 ml each; 10 ml for plasma, 8 ml for serum) will be obtained by venipuncture. The first sample is taken pre-immobilisation and the second one post-immobilisation. A total of 36 ml of venous blood will be taken over the entire study period. Aliquots of plasma and serum will be frozen in liquid nitrogen and stored at –80°C until analysis.

7.1.4 Muscle biopsies

A single muscle biopsy will be obtained from the designated leg to be immobilized during the pre and post immobilisation test days. Per subject we will take a total of two muscle tissue samples. Biopsies will be obtained from the middle region of the *m. vastus lateralis* muscle (15 cm above the patella) and approximately 2 cm below entry through the fascia by the percutaneous needle biopsy technique described by Bergström et al. [50], using a modified needle (Maastricht Instruments). With this instrument, better results are obtained for sample size, and subjects' discomfort is minimized. In short, skin and muscle fascia will be locally anesthetized using 1% xylocaine. After 10 minutes a small incision will be made in the skin and fascia after which the biopsy needle will be introduced in the muscle. Vacuum will be applied to the needle and, with the needle kept *in situ*; a small muscle sample (total ± 70-150

mg) will be taken. Following the muscle biopsy, the skin will be closed using a Steristrip® and covered by Tegaderm® after which a pressure bandage will be applied (Acrylastic®).

The percutaneous needle biopsy technique was introduced within the Department of Human Movement Sciences by Profs. Hans Keizer and Harm Kuipers more than 25 years ago. Since this introduction, the technique has been successfully used to perform numerous human *in vivo* studies focusing on skeletal muscle metabolism within our Department (for review see [78, 79]). In addition, recently approved MEC proposals continue to use the muscle biopsy technique in young (MEC 09-3-080 MEC 11-3-073 MEC 12-3-063) and elderly men (MEC 07-3-086, MEC 12-3-030, MEC 13-3-024). Based on our extensive previous experience, discomfort for the subjects with the technique used in our laboratory is limited.

7.1.5 Other study parameters

Other study parameters include plasma glucose and insulin concentrations, age, body weight, body height, BMI and blood pressure.

7.2 Randomization, blinding and treatment allocation

In this study, all eligible subjects will be allocated (1:1 ratio) to the ND or the LEU group in a randomized manner. Based on the order in which subjects enter the study, a unique participant code is assigned. These participant codes are linked to either the ND group or LEU group using a computer random number generator. All randomization procedures will be executed by an independent scientist. Supplement capsules and vials will be coded with the unique participant codes. A random number generator will also be used by an independent scientist when designating a leg for immobilisation. As said, data from placebo group from parallel running study (Creation, METC 13-3-023) will be used.

7.3 Study procedures

7.3.1 Screening & pre-testing

- Medical questionnaire will be filled in.
- Body weight and height will be measured.
- Blood pressure will be determined.
- 1-RM will be estimated.
- Use of crutches will be explained and practiced.

7.3.2 Diet and activity

- Subjects will consume a standardized meal (*Aviko maaltijdpannetje*) the evening before the test day.
- All volunteers will be instructed to refrain from any sort of heavy physical exercise 2 d before the first test day and during the study until the last visit
- All volunteers will be instructed to keep their diet as constant as possible during the intervention. A constant diet should be maintained to avoid any unanticipated effects of diet on the measured. Subjects will be asked to record their food intake and physical activity for 3 days before the start of the test day.

7.3.3 Experimental test days (2x)

- Subjects will report to the laboratory in the morning after an overnight fast
- A CT-scan will be performed as described previously [80].
- A single blood draw and muscle sample will be obtained.
- 1-RM will be tested

Please see section 3 and Figure 1, for a total overview of the study protocol.

7.3.4 Analyses

Quadriceps muscle CSA will be determined from CT-scans, as described previously [81].

Blood samples used for plasma will be collected in EDTA containing tubes and centrifuged at 1000 g and 4°C for 10 min. Blood samples used for serum will be allowed to clot for 30min in vertical position at room temperature and the centrifuged at 1100-1300 g and 4°C for 10 min. Aliquots of plasma and serum will be frozen in liquid nitrogen and stored at –80°C until analysis of blood serum/plasma concentrations of nandrolone, testosterone, CRP, IGF-I, IGFBP-3, insulin, glucose, vitamin D, cortisol, hGH and C-peptide will be measured by means of hormone assays. The remainder of the aliquots (if applicable) will be stored for any additional measurements.

Muscle samples will be cut into 5 µm thick cross sections and will be stained for immuno-histochemical determination of fiber type distribution, fiber type specific CSA, myonuclear content and satellite cell content [80, 82]. The latter will provide necessary information on the cellular mechanisms underlying the potential reduction in muscle mass. In addition, muscle samples will be analysed by the Western blotting technique, as described previously [83]. The expression and phosphorylation status of proteins from the mTOR pathway (i.e. PKB/Akt, mTOR, 4E-BP1, p70S6k, S6, TSC1/2) will be determined as well as expression of

the myogenic regulatory factors (i.e. MyoD, Myogenin, MRF4 and Myf5), AMPK, eukaryotic initiation factor 2 (eIF2), ERK1/2, mdm2, Numb (22700758), anti–androgen receptor (AR), IGF-I and myostatin. Poly- and monoclonal primary antibodies will be purchased from Cell Signalling Technologies (Beverly, MA, USA), and Santa Cruz Biotechnology (California, USA). Quantitative Real-Time PCR Analysis of MAFbx/Atrogin-1, MuRF1, FoxO and Ubiquitin Expression will be done. Activation of NF-κB will be measured in serum by the ELISA technique. Antibody against Pax7 and immunocytochemical techniques will be used to identify satellite cells and myonuclei. Histological analysis will require approximately 20-30mg of muscle tissue. Western blotting and RT-PCR will require ±50mg and 20mg of muscle tissue, respectively. Any remaining muscle tissue will be stored for possible future analyses or re-analysis.

Total energy intake and macronutrient composition of the diet will be determined from the dietary intake records. In addition, physical activity level will be assessed by analysing the physical activity diaries.

7.4 Withdrawal of individual subjects

Subjects can leave the study at any time for any reason if they wish to do so without any consequences. The investigator can decide to withdraw a subject from the study for urgent medical reasons or noncompliance to the protocol.

7.5 Replacement of individual subjects after withdrawal

In case of withdrawal, the subject will not be replaced. A 10% drop out rate has been included in the sample size calculation.

7.6 Follow-up of subjects withdrawn from treatment

Withdrawing of a subject does not need any form of explanation by the subject. All subjects in this study are included for follow-up after withdrawal of the study. A final follow-up call will be made 6 weeks after withdrawal to check whether full recovery has taken place.

7.7 Premature termination of the study

Severe physical discomfort caused by the study, noticed by the principal investigator, coordinating investigator or subjects themselves will lead to immediate termination of the study.

8. SAFETY REPORTING

Section 10 WMO event

In accordance to section 10, subsection 1, of the WMO, the investigator will inform the subjects and the reviewing accredited METC if anything occurs, on the basis of which it appears that the disadvantages of participation may be significantly greater than was foreseen in the research proposal. The study will be suspended pending further review by the accredited METC, except insofar as suspension would jeopardise the subjects' health. The investigator will take care that all subjects are kept informed.

8.1 Adverse and serious adverse events

Adverse events are defined as any undesirable experience occurring to a subject during the study, whether or not considered related to the investigational product. All adverse events reported spontaneously by the subject or observed by the investigator or his staff will be recorded.

A serious adverse event is any untoward medical occurrence or effect that at any dose:

- results in death;
- is life threatening (at the time of the event);
- requires hospitalisation or prolongation of existing inpatients' hospitalisation;
- results in persistent or significant disability or incapacity;
- is a congenital anomaly or birth defect;
- is a new event of the trial likely to affect the safety of the subjects, such as an unexpected outcome of an adverse reaction, lack of efficacy of an IMP used for the treatment of a life threatening disease, major safety finding from a newly completed animal study, etc.

All SAEs that occur during the study will be reported through the web portal *ToetsingOnline* to the accredited METC that approved the protocol, within 15 days after the sponsor has first knowledge of the serious adverse reactions.

SAEs that result in death or are life threatening should be reported expedited. The expedited reporting will occur not later than 7 days after the responsible investigator has first knowledge of the adverse reaction. This is for a preliminary report with another 8 days for completion of the report.

8.2 Follow-up of adverse events

All adverse events will be followed until they have abated, or until a stable situation has been reached. Depending on the event, follow up may require additional tests or medical procedures as indicated, and/or referral to the general physician or a medical specialist. SAE's need to be reported till the end of study in the Netherlands, as defined in the protocol.

9. STATISTICAL ANALYSIS

All data will be presented as means ± SEM. The baseline values of the two groups (i.e. weight, height, BMI, body composition, strength, quadriceps CSA) will be compared independently against one another (i.e. ND vs Leu vs placebo from MEC 13-3-023) using a one way ANOVA to ensure that they were correctly randomized and that no baseline differences exist. Differences in quadriceps CSA, muscle fibre size, satellite cell content and strength between pre- and post-immobilisation measurements will be analysed using a repeated measures analysis of variance (ANOVA) with time (pre and post) as within-subjects factor and treatment (ND vs Leu vs placebo from MEC 13-3-023) as between-subjects factor. Similarly, repeated measures ANOVA with multiple time points (i.e. pre-immobilisation and post-immobilisation) will be used to determine changes over time in muscle and/or blood parameters. In case of > 2 dropouts or missing values we will use linear mixed models; missing values will not be replaced manually. Should there be any significant differences between groups at baseline we will use ANCOVA with the baseline characteristics as covariates. Statistical significance will be set at $P<0.05$. Data will be analysed with IBM SPSS version 19.0.

10. ETHICAL CONSIDERATIONS

10.1 Regulation statement

This study will be conducted according to the principles of the declaration of Helsinki (Brazil, 2013) and in accordance with the Medical Research Involving Human Subjects Act (WMO).

10.2 Recruitment and consent

All subjects will be recruited through flyers situated around the campus and on dedicated bulletin boards within the University of Maastricht. In order to ascertain that the participants in this study are well informed, both written and oral explanation of the procedures of this study will be provided by the coordinating investigator. After all information is provided, subjects will be given at least one week to consider their decision before their informed consent is obtained. An independent MD (F. Hartgens) will be assigned to this project, who can be contacted by the subjects in case they want further information.

10.3 Benefits and risks assessment, group relatedness

The risks involved in participating in this experiment are minimal. If the subjects are interested, they receive their own results from the tests. The subjects receive a reimbursement for the time investment and burden of the study. Besides this, there is no additional benefit for participating in this study.

The incision made for obtaining the muscle biopsy will be performed by an experienced physician and will heal completely. Within our research group we have extensive experience with taking muscle biopsies. There is a small risk of infection and the muscle biopsy can in lead to minor hematoma. During the blood draw there is a small risk of fainting or haematoma. These risks are minimized by using trained and experienced personnel for taking the muscle biopsies and blood draws. Adequate pressure will be applied after the blood draw and biopsies to minimise the risk of hematoma.

The Aviko vacuum-packed and pre-weighed meals are normal food products and have been cleared for human consumption. There are no complications associated with the procedure of a single slice lower limb CT scan. The level of radiation emitted is very low; approximately 0.053 mSv per scan. In this study, subjects will undergo two CT scans and will therefore be subjected to 0.106 mSV of radiation. These scans are done routinely in studies by our group (e.g. see MEC 06-3-062, 11-3-073, 12-3-012). For comparison, the present study will provide a 25 times lower dose of radiation than the ordinary background radiation in the Netherlands (i.e. 2.5 mSv/year) and a similar level to that which one would be exposed to during a two week winter sports holiday.

In terms of time investment, the screening visit will take 1.5-2 hours of the subject's time, the other visits will take ~2.5 hours for the test days and ~1 hour for the casting day. An additional burden will be the requirement for subjects to fill in a 3 day food and activity diary (see appendix F2.1 and F2.2) before the pre and post immobilisation visits. However, the greatest inconvenience in terms of time and also mobility will be the 7 days of one-legged immobilisation.

One leg will be immobilized for seven days by means of a full leg cast at a 30 degree angle of flexion (**see Figure 2**). In a healthy population deep vein thrombosis (DVT) will develop in 1 out every 1000 individuals a year [84]. After surgery and/or bone fracture immobilisation of two or more joints will increase the risk of developing DVT. The incidence of DVT occurrence during limb immobilisation following surgery and/or bone fracture is estimated between ~0.2 and ~17% [55, 85-88]. However, in the present study prophylactic medication to prevent DVT is not warranted when a single joint is immobilized [55, 85], particularly for a time period of only 7 days. However, to further minimize the risk of DVT development, subjects with any family history of thrombosis will be excluded (please see exclusion criteria; section 4.3) and all subjects will perform daily exercises to activate the calf muscle pump. The time needed to perform these exercises is 5 minutes. Exercises will be performed 3 times a day (i.e. morning, afternoon and evening) for the 7 day immobilisation period. Finally, throughout the immobilisation period all subjects will be monitored closely by the research team, who will establish regular contact with the subjects, to identify early signs of DVT development and act accordingly. Thus, in this study the overall risk of DVT is minimal (<0.1%).

The leg cast will cause a large degree of physical impairment. Subjects will be provided with crutches because they are not allowed to bear any weight on their immobilized leg. Full instructions will be given by a physiotherapist on the correct use of crutches. To minimize the risk of any injury due to this loss of physical mobility, activities like driving a car and/or bicycle, and sport activities will be prohibited for the 7 day immobilisation period. Other activities might require assistance (e.g. travelling, climbing stairs). Walking is only permitted with crutches, without weight bearing on the immobilized leg. During the first days/week after removal of the leg cast subjects will probably encounter the effects of the reduced muscle mass and strength of their immobilized leg. Normal mobility and activities of daily living will no longer be impaired, but sports performance might be somewhat below normal level in the first week after the study. However, since only healthy subjects will participate in this study, muscle function will most likely completely restore itself within a few weeks after cast removal [52, 79]. Indeed, it has been shown that even after 3 weeks of leg immobilisation in young individuals, the resumption of spontaneous activity for 2 weeks results in the recovery of 90%

of muscle strength and 95% of muscle fibre size [52]. A final follow-up phone call will be performed 6 weeks after cast removal to confirm that full recovery has taken place. If this is not the case, further follow up will be performed or referral to the general practitioner will take place when appropriate.

As discussed above, the use of 200 mg nandrolone decanoate is generally accepted in the Netherlands and higher doses have been safely used for longer periods in time, even in patients [41{Chlebowski, 1986 #56, 64]. The leucine supplement is widely accepted for scientific and clinical purpose. No major risks are associated with the use of leucine, even for longer periods or when using higher dosages than in the current study, in healthy volunteers. More detailed information is included in paragraph 12.

10.4 Compensation for injury

The sponsor/investigator has a liability insurance which is in accordance with article 7, subsection 6 of the WMO.

The sponsor (also) has an insurance which is in accordance with the legal requirements in the Netherlands (Article 7 WMO and the Measure regarding Compulsory Insurance for Clinical Research in Humans of 23th June 2003). This insurance provides cover for damage to research subjects through injury or death caused by the study.

1. € 450.000,-- (i.e. four hundred and fifty thousand Euro) for death or injury for each subject who participates in the Research;
2. € 3.500.000,-- (i.e. three million five hundred thousand Euro) for death or injury for all subjects who participate in the Research;
3. € 5.000.000,-- (i.e. five million Euro) for the total damage incurred by the organisation for all damage disclosed by scientific research for the Sponsor as ‘verrichter’ in the meaning of said Act in each year of insurance coverage.

The insurance applies to the damage that becomes apparent during the study or within 4 years after the end of the study.

10.5 Incentives (if applicable)

Subjects will receive €225 for completion of the study. Travel expenses will be covered separately. If the subject does not complete the entire study, he will receive payment according to ratio of the study completed. If the subject drops out after the screening but before the start of the test day, he will not receive any compensation (except for travel expenses).

11. ADMINISTRATIVE ASPECTS AND PUBLICATION

11.1 Handling and storage of data and documents

To protect the privacy of the participants, all collected data will be encoded numerically (i.e. not directly traceable to the subjects) and only the principal investigators will have access to the encoding key and the acquired data. If required, the data can be accessed by the following independent organizations: the Medical Ethical Committee, a monitor from the CTCM or The Health Care Inspectorate. All subjects will be offered full access to their own data and the outcomes of the project will be explained to them. The CT scans will only be analysed in the context of the present study (i.e. to determine muscle CSA) and as such, potential abnormalities may be missed.

All subject data will be kept for 15 years, and (with approval of the subject), all human material will also be stored for 15 years for potential future analysis in the line of this research, after which all material will be destroyed.

11.2 Amendments

All amendments to the protocol (i.e. regarding the intervention itself as well as the different measurements and/or tests performed) will be notified to the METC. Substantial amendments will be submitted for approval before they will be implemented in the study protocol. Annual reports will be submitted to the METC regarding progress of the study, number of subjects included/completed, and serious adverse events. Upon ending of the study, the METC will be notified within 8 weeks.

11.3 Annual progress report

The sponsor/investigator will submit a summary of the progress of the trial to the accredited METC once a year. Information will be provided on the date of inclusion of the first subject, numbers of subjects included and numbers of subjects that have completed the trial, serious adverse events/ serious adverse reactions, other problems, and amendments.

11.4 End of study report

The investigator will notify the accredited METC of the end of the study within a period of 8 weeks. The end of the study is defined as the last subject’s last visit.

In case the study is ended prematurely, the investigator will notify the accredited METC within 15 days, including the reasons for the premature termination.

Within one year after the end of the study, the investigator/sponsor will submit a final study report with the results of the study, including any publications/abstracts of the study, to the accredited METC.

11.1 Public disclosure and publication policy

The results of this investigation will be published in a high-impact, scientific journal, regardless of the outcome of this study. All subjects will be given insight into the (individual) results of this study.

12. STRUCTURED RISK ANALYSIS

12.1 Potential issues of concern

Bagchus et al (2006) described safety parameters of nandrolone decanoate and reported the following drug-related adverse events (AEs): ”testicular pain (one subject), injection site pain (one subject), injection site reaction (one subject), back pain (one subject), fatigue (five subjects), hot flushes (one subject), and leg pain (one subject). The intensity of all drug-related AEs was assessed as mild. Local tolerance was good. No itching, swelling, or bruising was observed after injection in the gluteal muscle. Two subjects showed mild redness directly after dosing. Pain at the injection site was reported in 13 subjects 2 h after injection. Mild pain at the injection site was still present in one subject in each dose group 24 h after injection.”

There have been no reported serious issues with leucine supplementation.

12.2 Synthesis

No serious issues have been reported with ND injection or leucine supplementation. The intensity of all drug-related adverse events in Bagchus’ study was assessed as mild Problems commonly associated with ND supplementation are largely based on anecdotal evidence and there is no consistent evidence in previous studies that these side-effects are common. We use 200 mg to avoid any problems potentially associated with large single doses of ND. This dosage has been used previously in several studies without any negative side effects, with the exception of the study mentioned above (ref). Some studies have used even higher daily dosages without problems. We will exclude people that have contraindications according to the instructions of ND. Furthermore, the relative short-term nature of the present study (with only 1 week in total of supplementation) is unlikely to cause any serious complaints such as sleep problems or aggressive behaviour. Any complaints occurring during the study will be monitored thoroughly.

13. REFERENCES

1. Deitrick, J.E., *The effect of immobilization on metabolic and physiological functions of normal men.* Bull N Y Acad Med, 1948. **24**(6): p. 364-75.
2. Gibson, J.N., et al., *Decrease in human quadriceps muscle protein turnover consequent upon leg immobilization.* Clin Sci (Lond), 1987. **72**(4): p. 503-9.
3. Stuart, C.A., et al., *Bed-rest-induced insulin resistance occurs primarily in muscle.* Metabolism, 1988. **37**(8): p. 802-6.
4. Tzankoff, S.P. and A.H. Norris, *Effect of muscle mass decrease on age-related BMR changes.* J Appl Physiol Respir Environ Exerc Physiol, 1977. **43**(6): p. 1001-6.
5. Adams, G.R., V.J. Caiozzo, and K.M. Baldwin, *Skeletal muscle unweighting: spaceflight and ground-based models.* J Appl Physiol (1985), 2003. **95**(6): p. 2185-201.
6. Appell, H.J., *Muscular atrophy following immobilisation. A review.* Sports Med, 1990. **10**(1): p. 42-58.
7. Berg, H.E., et al., *Effects of lower limb unloading on skeletal muscle mass and function in humans.* J Appl Physiol (1985), 1991. **70**(4): p. 1882-5.
8. Blakemore, S.J., et al., *Effects of limb immobilization on cytochrome c oxidase activity and GLUT4 and GLUT5 protein expression in human skeletal muscle.* Clin Sci (Lond), 1996. **91**(5): p. 591-9.
9. Christensen, B., et al., *Short-term immobilization and recovery affect skeletal muscle but not collagen tissue turnover in humans.* J Appl Physiol (1985), 2008. **105**(6): p. 1845-51.
10. Hespel, P., et al., *Oral creatine supplementation facilitates the rehabilitation of disuse atrophy and alters the expression of muscle myogenic factors in humans.* J Physiol, 2001. **536**(Pt 2): p. 625-33.
11. White, M.J., C.T. Davies, and P. Brooksby, *The effects of short-term voluntary immobilization on the contractile properties of the human triceps surae.* Q J Exp Physiol, 1984. **69**(4): p. 685-91.
12. Dirks, M.L., et al., *Neuromuscular electrical stimulation prevents muscle disuse atrophy during leg immobilization in humans.* Acta Physiol (Oxf), 2014. **210**(3): p. 628-41.
13. Anthony, J.C., T.G. Anthony, and D.K. Layman, *Leucine supplementation enhances skeletal muscle recovery in rats following exercise.* J Nutr, 1999. **129**(6): p. 1102-6.
14. Anthony, T.G., et al., *Oral administration of leucine stimulates ribosomal protein mRNA translation but not global rates of protein synthesis in the liver of rats.* J Nutr, 2001. **131**(4): p. 1171-6.
15. Koopman, R., et al., *Combined ingestion of protein and free leucine with carbohydrate increases postexercise muscle protein synthesis in vivo in male subjects.* Am J Physiol Endocrinol Metab, 2005. **288**(4): p. E645-53.
16. Garlick, P.J., *The role of leucine in the regulation of protein metabolism.* J Nutr, 2005. **135**(6 Suppl): p. 1553S-6S.
17. Nair, K.S., R.G. Schwartz, and S. Welle, *Leucine as a regulator of whole body and skeletal muscle protein metabolism in humans.* Am J Physiol, 1992. **263**(5 Pt 1): p. E928-34.
18. van Loon, L.J., et al., *Amino acid ingestion strongly enhances insulin secretion in patients with long-term type 2 diabetes.* Diabetes Care, 2003. **26**(3): p. 625-30.
19. Anthony, J.C., et al., *Contribution of insulin to the translational control of protein synthesis in skeletal muscle by leucine.* Am J Physiol Endocrinol Metab, 2002. **282**(5): p. E1092-101.
20. Anthony, J.C., et al., *Orally administered leucine enhances protein synthesis in skeletal muscle of diabetic rats in the absence of increases in 4E-BP1 or S6K1 phosphorylation.* Diabetes, 2002. **51**(4): p. 928-36.
21. Kimball, S.R. and L.S. Jefferson, *Regulation of global and specific mRNA translation by oral administration of branched-chain amino acids.* Biochem Biophys Res Commun, 2004. **313**(2): p. 423-7.
22. Magne, H., et al., *Contrarily to whey and high protein diets, dietary free leucine supplementation cannot reverse the lack of recovery of muscle mass after prolonged immobilization during ageing.* J Physiol, 2012. **590**(Pt 8): p. 2035-49.

23. Magne, H., et al., *Unilateral hindlimb casting induced a delayed generalized muscle atrophy during rehabilitation that is prevented by a whey or a high protein diet but not a free leucine-enriched diet.* PLoS One, 2013. **8**(8): p. e70130.
24. Savary-Auzeloux, I., et al., *A dietary supplementation with leucine and antioxidants is capable to accelerate muscle mass recovery after immobilization in adult rats.* PLoS One, 2013. **8**(11): p. e81495.
25. Baptista, I.L., et al., *Leucine attenuates skeletal muscle wasting via inhibition of ubiquitin ligases.* Muscle Nerve, 2010. **41**(6): p. 800-8.
26. Maki, T., et al., *Branched-chain amino acids reduce hindlimb suspension-induced muscle atrophy and protein levels of atrogin-1 and MuRF1 in rats.* Nutr Res, 2012. **32**(9): p. 676-83.
27. Dardevet, D., et al., *Postprandial stimulation of muscle protein synthesis in old rats can be restored by a leucine-supplemented meal.* J Nutr, 2002. **132**(1): p. 95-100.
28. Katsanos, C.S., et al., *A high proportion of leucine is required for optimal stimulation of the rate of muscle protein synthesis by essential amino acids in the elderly.* Am J Physiol Endocrinol Metab, 2006. **291**(2): p. E381-7.
29. Rieu, I., et al., *Increased availability of leucine with leucine-rich whey proteins improves postprandial muscle protein synthesis in aging rats.* Nutrition, 2007. **23**(4): p. 323-31.
30. Rieu, I., et al., *Leucine supplementation improves muscle protein synthesis in elderly men independently of hyperaminoacidaemia.* J Physiol, 2006. **575**(Pt 1): p. 305-15.
31. Rieu, I., et al., *Leucine-supplemented meal feeding for ten days beneficially affects postprandial muscle protein synthesis in old rats.* J Nutr, 2003. **133**(4): p. 1198-205.
32. Bagchus, W.M., et al., *Pharmacokinetic evaluation of three different intramuscular doses of nandrolone decanoate: analysis of serum and urine samples in healthy men.* J Clin Endocrinol Metab, 2005. **90**(5): p. 2624-30.
33. Belkien, L., et al., *Pharmacokinetics of 19-nortestosterone esters in normal men.* J Steroid Biochem, 1985. **22**(5): p. 623-9.
34. Minto, C.F., et al., *Pharmacokinetics and pharmacodynamics of nandrolone esters in oil vehicle: effects of ester, injection site and injection volume.* J Pharmacol Exp Ther, 1997. **281**(1): p. 93-102.
35. Wijnand, H.P., A.M. Bosch, and C.W. Donker, *Pharmacokinetic parameters of nandrolone (19-nortestosterone) after intramuscular administration of nandrolone decanoate (Deca-Durabolin) to healthy volunteers.* Acta Endocrinol Suppl (Copenh), 1985. **271**: p. 19-30.
36. Oliveira, E.N., et al., *Nandrolone decanoate is able to modulate proliferation and adhesion of myoblasts.* Endocr Regul, 2014. **48**(3): p. 152-8.
37. Allouh, M.Z. and B.W. Rosser, *Nandrolone decanoate increases satellite cell numbers in the chicken pectoralis muscle.* Histol Histopathol, 2010. **25**(2): p. 133-40.
38. van Marken Lichtenbelt, W.D., et al., *Bodybuilders' body composition: effect of nandrolone decanoate.* Med Sci Sports Exerc, 2004. **36**(3): p. 484-9.
39. Storer, T.W., et al., *A randomized, placebo-controlled trial of nandrolone decanoate in human immunodeficiency virus-infected men with mild to moderate weight loss with recombinant human growth hormone as active reference treatment.* J Clin Endocrinol Metab, 2005. **90**(8): p. 4474-82.
40. Gold, J., et al., *Safety and efficacy of nandrolone decanoate for treatment of wasting in patients with HIV infection.* AIDS, 1996. **10**(7): p. 745-52.
41. Sattler, F.R., et al., *Effects of pharmacological doses of nandrolone decanoate and progressive resistance training in immunodeficient patients infected with human immunodeficiency virus.* J Clin Endocrinol Metab, 1999. **84**(4): p. 1268-76.
42. Schroeder, E.T., M. Terk, and F.R. Sattler, *Androgen therapy improves muscle mass and strength but not muscle quality: results from two studies.* Am J Physiol Endocrinol Metab, 2003. **285**(1): p. E16-24.
43. Strawford, A., et al., *Effects of nandrolone decanoate therapy in borderline hypogonadal men with HIV-associated weight loss.* J Acquir Immune Defic Syndr Hum Retrovirol, 1999. **20**(2): p. 137-46.

44. Creutzberg, E.C., et al., *A role for anabolic steroids in the rehabilitation of patients with COPD? A double-blind, placebo-controlled, randomized trial.* Chest, 2003. **124**(5): p. 1733-42.
45. Chlebowski, R.T., et al., *Influence of nandrolone decanoate on weight loss in advanced non-small cell lung cancer.* Cancer, 1986. **58**(1): p. 183-6.
46. Hassager, C., et al., *Nandrolone decanoate treatment of post-menopausal osteoporosis for 2 years and effects of withdrawal.* Maturitas, 1989. **11**(4): p. 305-17.
47. Bouhlel, A., W.H. Joumaa, and C. Leoty, *Nandrolone decanoate reduces changes induced by hindlimb suspension in voltage-dependent tension of rat soleus muscle.* Jpn J Physiol, 2003. **53**(2): p. 77-87.
48. Joumaa, W.H., et al., *Nandrolone decanoate pre-treatment attenuates unweighting-induced functional changes in rat soleus muscle.* Acta Physiol Scand, 2002. **176**(4): p. 301-9.
49. Wall, B.T. and L.J. van Loon, *Nutritional strategies to attenuate muscle disuse atrophy.* Nutr Rev, 2013. **71**(4): p. 195-208.
50. Bergstrom, J., *Percutaneous needle biopsy of skeletal muscle in physiological and clinical research.* Scand J Clin Lab Invest, 1975. **35**(7): p. 609-16.
51. Verdijk, L.B., et al., *One-repetition maximum strength test represents a valid means to assess leg strength in vivo in humans.* J Sports Sci, 2009. **27**(1): p. 59-68.
52. Hortobagyi, T., et al., *Changes in muscle strength, muscle fibre size and myofibrillar gene expression after immobilization and retraining in humans.* J Physiol, 2000. **524 Pt 1**: p. 293-304.
53. Suetta, C., et al., *Ageing is associated with diminished muscle re-growth and myogenic precursor cell expansion early after immobility-induced atrophy in human skeletal muscle.* J Physiol, 2013. **591**(Pt 15): p. 3789-804.
54. Smith, G.I., et al., *Differences in muscle protein synthesis and anabolic signaling in the postabsorptive state and in response to food in 65-80 year old men and women.* PLoS One, 2008. **3**(3): p. e1875.
55. Metz, R., E.M.M. Verleisdonk, and G.J.M.G. van der Heijden, *Insufficient evidence for routine use of thromboprophylaxis in ambulatory patients with an isolated lower leg injury requiring immobilization: results of a mete-analysis.* Eur J Trauma Emerg Surg, 2009. **35**: p. 169-175.
56. Berg, H.E., et al., *Effects of lower limb unloading on skeletal muscle mass and function in humans.* J Appl Physiol, 1991. **70**(4): p. 1882-5.
57. Haegens, A., et al., *Leucine induces myofibrillar protein accretion in cultured skeletal muscle through mTOR dependent and -independent control of myosin heavy chain mRNA levels.* Mol Nutr Food Res, 2012. **56**(5): p. 741-52.
58. Borsheim, E., et al., *Effect of amino acid supplementation on muscle mass, strength and physical function in elderly.* Clin Nutr, 2008. **27**(2): p. 189-95.
59. van Loon, L.J., *Leucine as a pharmaconutrient in health and disease.* Curr Opin Clin Nutr Metab Care, 2012. **15**(1): p. 71-7.
60. Leenders, M., et al., *Prolonged leucine supplementation does not augment muscle mass or affect glycemic control in elderly type 2 diabetic men.* J Nutr, 2011. **141**(6): p. 1070-6.
61. Verhoeven, S., et al., *Long-term leucine supplementation does not increase muscle mass or strength in healthy elderly men.* Am J Clin Nutr, 2009. **89**(5): p. 1468-75.
62. Friedl, K.E., et al., *Comparison of the effects of high dose testosterone and 19-nortestosterone to a replacement dose of testosterone on strength and body composition in normal men.* J Steroid Biochem Mol Biol, 1991. **40**(4-6): p. 607-12.
63. Joumaa, W.H. and C. Leoty, *Differential effects of nandrolone decanoate in fast and slow rat skeletal muscles.* Med Sci Sports Exerc, 2001. **33**(3): p. 397-403.
64. Mulligan, K., et al., *Effect of nandrolone decanoate therapy on weight and lean body mass in HIV-infected women with weight loss: a randomized, double-blind, placebo-controlled, multicenter trial.* Arch Intern Med, 2005. **165**(5): p. 578-85.
65. Hartgens, F., et al., *Body composition and anthropometry in bodybuilders: regional changes due to nandrolone decanoate administration.* Int J Sports Med, 2001. **22**(3): p. 235-41.

66. Fahey, T.D. and C.H. Brown, *The effects of an anabolic steroid on the strength, body composition, and endurance of college males when accompanied by a weight training program.* Med Sci Sports, 1973. **5**(4): p. 272-6.
67. Tamaki, T., et al., *Anabolic steroids increase exercise tolerance.* Am J Physiol Endocrinol Metab, 2001. **280**(6): p. E973-81.
68. Gerber, C., et al., *Anabolic steroids reduce muscle damage caused by rotator cuff tendon release in an experimental study in rabbits.* J Bone Joint Surg Am, 2011. **93**(23): p. 2189-95.
69. Hohmann, E., et al., *Anabolic steroids after total knee arthroplasty. A double blinded prospective pilot study.* J Orthop Surg Res, 2010. **5**: p. 93.
70. Piovesan, R.F., et al., *Effect of nandrolone decanoate on skeletal muscle repair.* Int J Sports Med, 2013. **34**(1): p. 87-2.
71. Tamaki, T., et al., *Anabolic-androgenic steroid does not enhance compensatory muscle hypertrophy but significantly diminish muscle damages in the rat surgical ablation model.* Histochem Cell Biol, 2009. **132**(1): p. 71-81.
72. Batterham, M.J. and R. Garsia, *A comparison of megestrol acetate, nandrolone decanoate and dietary counselling for HIV associated weight loss.* Int J Androl, 2001. **24**(4): p. 232-40.
73. Schols, A.M., et al., *Physiologic effects of nutritional support and anabolic steroids in patients with chronic obstructive pulmonary disease. A placebo-controlled randomized trial.* Am J Respir Crit Care Med, 1995. **152**(4 Pt 1): p. 1268-74.
74. Velema, M.S., B.H. Kwa, and W. de Ronde, *Should androgenic anabolic steroids be considered in the treatment regime of selected chronic obstructive pulmonary disease patients?* Curr Opin Pulm Med, 2012. **18**(2): p. 118-24.
75. Johansen, J.S., et al., *Treatment of postmenopausal osteoporosis: is the anabolic steroid nandrolone decanoate a candidate?* Bone Miner, 1989. **6**(1): p. 77-86.
76. Johansen, K.L., K. Mulligan, and M. Schambelan, *Anabolic effects of nandrolone decanoate in patients receiving dialysis: a randomized controlled trial.* JAMA, 1999. **281**(14): p. 1275-81.
77. Roman, E., et al., *Randomized pilot study: effects of an exercise programme and leucine supplementation in patients with cirrhosis.* Dig Dis Sci, 2014. **59**(8): p. 1966-75.
78. Koopman, R., et al., *Nutritional interventions to promote post-exercise muscle protein synthesis.* Sports Med, 2007. **37**(10): p. 895-906.
79. Koopman, R. and L.J.C. van Loon, *Aging, exercise and muscle protein metabolism.* J Appl Physiol, 2009.
80. Verdijk, L., et al., *Skeletal muscle hypertrophy following resistance training is accompanied by a fiber type-specific increase in satellite cell content in elderly men.* J Gerontol A Biol Sci Med Sci, 2009. **64**(3): p. 332-9.
81. Leenders, M., et al., *Protein Supplementation During Resistance-Type Exercise Training in the Elderly.* Med Sci Sports Exerc, 2012.
82. Verdijk, L.B., et al., *Skeletal muscle hypertrophy following resistance training is accompanied by a fiber type-specific increase in satellite cell content in elderly men* J Gerontol A Biol Sci Med Sci, 2009.
83. Koopman, R., et al., *Protein ingestion further augments S6K1 phosphorylation in skeletal muscle following resistance type exercise in males.* J Nutr, 2007. **137**(8): p. 1880-6.
84. Ageno, W., et al., *Epidemiology and risk factors of venous thromboembolism.* Semin Thromb Hemost, 2006. **32**(7): p. 651-8.
85. Glover, E., et al., *Immobilization induces anabolic resistance in human myofibrillar protein synthesis with low and high dose amino acid infusion.* J Physiol, 2008. **586**: p. 6049-61.
86. Ettema, H.B., et al., *Prevention of venous thromboembolism in patients with immobilization of the lower extremities: a meta-analysis of randomized controlled trials.* J Thromb Haemost, 2008. **6**(7): p. 1093-8.
87. Nokes, T.J. and J. Keenan, *Thromboprophylaxis in patients with lower limb immobilisation - review of current status.* Br J Haematol, 2009.

88. Testroote, M., et al., *Low molecular weight heparin for prevention of venous thromboembolism in patients with lower-leg immobilization.* Cochrane Database Syst Rev, 2008(4): p. CD006681.
